# Unveiling the dynamics of team cognition in emergency response teams

**DOI:** 10.3389/fpsyg.2025.1534224

**Published:** 2025-03-26

**Authors:** Reza Esmaeili, Mohammad Yazdi, Masoud Rismanchian, Mahnaz Shakerian

**Affiliations:** ^1^Student Research Committee, Department of Occupational Health and Safety Engineering, School of Health, Isfahan University of Medical Sciences, Isfahan, Iran; ^2^School of Computing, Engineering & Physical Sciences, University of the West of Scotland (UWS), London, United Kingdom; ^3^Faculty of Science and Engineering, Macquarie University, Sydney, NSW, Australia; ^4^Department of Occupational Health and Safety Engineering, School of Health, Isfahan University of Medical Sciences, Isfahan, Iran

**Keywords:** team cognition, emergency response team, meta-synthesis, weighting, modeling

## Abstract

**Background:**

Effective emergency response in various industries depends on the synergy between team coordination and cognitive abilities. Industries should prioritize investing in the development of team cognition to improve readiness and ensure swift, effective responses to emergencies and crises. This study aimed to identify and model factors influencing team cognition within Emergency Response Teams (ERTs).

**Methods:**

This cross-sectional study undertook two principal phases: qualitative research using meta-synthesis and quantitative research using Best Worst Method (BWM), Interpretive Structural Modeling (ISM), and Fuzzy Cognitive Mapping (FCM). These methods were employed to assign weights to factors, establish their hierarchy, and determine cause-and-effect relationships among team cognition shaping factors (TCSFs).

**Results:**

Through a comprehensive evaluation of the articles, 13 dimensions were identified as the primary TCSFs influencing team cognition. The reliability of the extracted factors was validated using the Kappa indicator, with a value of 0.63 signifying an acceptable level of agreement. Using BWM analysis, “Team maturity (The team members’ harmonization)” and “Inefficient 4Cs (communication, coordination, cooperation, and collaboration)” were identified as the most influential factors shaping team cognition, with weights of 0.132 and 0.112, respectively. ISM analysis revealed “Improper team training programs” as a critical independent factor influencing other dimensions. FCM modeling further emphasized the significance of “Failure in decision-making” and “Leadership behavior and performance” as pivotal contributors to team cognition, with “Team maturity” and “Inefficient 4Cs” achieving the highest centrality scores of 13.44 and 13.28, respectively.

**Conclusion:**

Stakeholders can enhance team performance and effectiveness in emergency situations by understanding the relative importance of various factors, their hierarchical relationships, and the causal links between them. This allows for informed decision-making and targeted interventions, such as training programs to improve team maturity and team communication.

## 1 Introduction

In emergency situations, it’s essential for team members to not only grasp their own roles but also understand the roles and responsibilities of their fellow team members within ERTs (Patrício and Franco, 2022). Clearly outlining these responsibilities is crucial for preventing chaos and ensuring effective command and coordination during relief efforts (Moon et al., 2020). ERTs often include a range of specialists, such as hazardous materials specialists, logistics specialists, and fire chiefs. The decision to employ such diverse expertise in ERTs reflects the increased complexity of incidents, higher expectations, and evolving industry standards, all of which introduce challenges related to human factors (Lin et al., 2020). Prior studies have explored various aspects of human factors in emergency response, including cooperative and competitive behavior, stress levels, perceived workload, team member reliability, and cognitive impairments (Nicoletti and Padovano, 2019; Magoua and Li, 2023; Wang et al., 2021). Thus, understanding the interplay between human behavior and the operational dynamics of ERTs during crises is vital for effective emergency preparedness and response. Given the frequency of natural and man-made disasters that lead to significant damage worldwide, real-time decision-making is critical during the initial response phase (Tatham et al., 2017). First responders, who come from diverse backgrounds like fire, medical, law enforcement, or public works teams, must work together as an *ad hoc* team to preserve lives and protect vulnerable infrastructure (White et al., 2023). For successful accountability operations, it’s important to understand cognition—not only at the individual level but also at the team level (Curseu et al., 2015). However, the concept of team cognition remains relatively underexplored in the literature on emergency response.

In the context of industrial work processes, an essential aspect of teamwork is understanding collective or team cognitive performance (Hagemann and Kluge, 2017). This multi-level phenomenon serves as a mechanism through which team members process information, make decisions, plan, learn, and adapt to changing conditions (Kazi et al., 2021). Team cognition involves elucidating the multi-level interactions between intra-individual and interpersonal cognitive processes (Fernandez et al., 2017). Essentially, it describes a state that promotes and enhances effective team performance, much like individual cognition, wherein key information necessary for predicting and executing tasks is organized, displayed, and distributed efficiently (DeChurch and Mesmer-Magnus, 2010). Team cognition provides a comprehensive framework that explains how knowledge is collectively distributed among team members. This shared knowledge enables team members to interpret information similarly, fostering common expectations and facilitating coordinated actions (Sætrevik, 2015).

High-stress, high-stakes work environments highlight the importance of team cognition, especially with significant workloads and severe repercussions for errors (Wildman et al., 2014). Team cognition’s key concepts are Team Mental Models (TMM), Transactive Memory Systems (TMS), and Team Situational Awareness (TSA) (Ellwart and Antoni, 2017). TMM measures the shared understanding of tasks, goals, and the work environment among team members. This collective understanding includes knowledge regarding team equipment, task requirements, how the team interacts, and each team member’s abilities (Santos et al., 2015). Cannon-Bowers et al. (1990) originally introduced the concept, emphasizing its importance for effective team coordination, particularly under pressure and time constraints. Research like Mohammed et al. (2010) demonstrates that TMM improve team anticipation and response to rapid change, leading to fewer errors and better performance over time. Despite this, a considerable portion of this research has mainly concentrated on controlled or static environments, resulting in a significant lack of understanding concerning the development and adaptation of TMMs in the unpredictable and rapid environments of ERTs. TMS emphasizes knowledge distribution and organization within teams, detailing information storage, sharing, and access via collaborative methods (Ali et al., 2021). Wegner (1987) introduced TMS, describing them as cognitive systems that boost team memory via specialization and trust among team members. According to Majchrzak et al. (2007), TMS improves emergency response coordination, task allocation, and expertise utilization. Despite the significance of these findings, the impact of real-world challenges (stress, time pressure, team composition) on TMS effectiveness in high-stakes emergencies remains largely unstudied. TSA reflects the team’s shared understanding of their current situation and their ability to predict future events (Stanton, 2016). The groundwork for understanding situational awareness, established by Endsley (1995), was later extended to team dynamics by Seppänen and Virrantaus (2015). TSA’s critical role in enhancing decision-making and accelerating response times in disaster relief was a key focus of their research. Simultaneously, they emphasized the considerable difficulties teams encounter when preserving TSA amidst high-pressure, dynamic circumstances. This highlights the necessity of additional research into strategies and mechanisms that can aid TSA within ERTs.

Individual constructs are well-understood, but integrative frameworks for their interaction during emergencies are underdeveloped. As an illustration, Plant and Stanton (2016) examined Distributed Situation Awareness (DSA) within search and rescue teams; however, they did not consider its links to Team Mental Models (TMM) or Transactive Memory Systems (TMS). van der Haar et al. (2015) also investigated communication’s contribution to TSA and TMS growth; however, their research did not consider contextual influences like organizational culture, team structure, and limited resources. This incremental approach unfortunately inhibits the development of a thorough understanding of how team cognition functions holistically within the dynamic and complex operational environments of ERTs.

### 1.1 Theoretical background

Studying team cognition presents organizations with valuable applications, particularly for response teams operating in emergency situations and crisis management. Research across various domains has examined this critical aspect. For example, Seppänen and Virrantaus (2015) emphasized the importance of TSA in disaster response operations, identifying proper information sharing as a key factor for improving TSA. Plant and Stanton (2016) highlighted the importance of Distributed Situation Awareness (DSA) and Distributed Cognition (DC) in Search and Rescue teams, pointing out that factors like information sharing, leadership performance, and adherence to procedures significantly impact these cognitive processes. Similarly, Sætrevik and Eid’s (2014) study on Emergency Response Teams (ERTs) suggested that the level of information sharing among team members reflects joint mental models and TSA, while team understanding and differing individual priorities play a role in shaping these constructs. In addition, Mohammed et al. (2015) highlighted the importance of TMM in enhancing team performance, with factors such as available time, shared knowledge, and team processes being critical for TMM formation. Van Der Haar et al. (2008) focused on the role of effective communication in crisis management teams, suggesting that fostering communication can be enhanced through the development of TMS and TSA. Moreover, Majchrzak et al. (2007) explored the significance of TMS as a framework for coordinating knowledge within teams, stressing its role in task assignment, managing member expertise credibility, and expertise coordination. Influential factors affecting TMS effectiveness included role ambiguity, trust, and training. Despite the extensive research conducted in the field of team cognition within emergency response and crisis management, there remains a gap in the literature regarding a comprehensive examination of the diverse factors influencing team cognition in this domain.

In recent years, researchers have significantly advanced our understanding of how team cognitive constructs such as TMM, TMS, and TSA influence team performance. However, many studies have looked at these constructs separately. For example, Fleştea et al. (2017) emphasized the role of TSA in improving team communication and coordination during disaster response. The research offers valuable insights into communication strategies, but its limited scope prevents a full examination of how TSA interacts with models such as TMM or TMS. Similarly, Curnin et al. (2015) studied DSA in multi-agency coordination but did not examine how it interacts with other cognitive processes in high-pressure situations. This fragmented approach leaves important gaps in understanding how these constructs function together, especially in emergency response teams (ERTs), where rapid decision-making under pressure is essential. Moreover, much of the current research is based on controlled simulations rather than real-world situations, which limits how applicable the findings are. For example, in their study on ERTs in a simulated environment, Mohammed et al. (2015) emphasized the use of TMM in improving the overall state of team cognition and improving team performance. However, they considered one of the limitations of their study to be the failure to evaluate the factors studied in a real environment (Mohammed et al., 2015). Another challenge is that factors like cultural differences, organizational structures, and team composition have not been explored in depth, even though they can significantly affect how well team cognition frameworks work (Laurila-Pant et al., 2023). To bridge these gaps, a more holistic approach is needed—one that captures the complex and evolving nature of real-world emergency situations.

Today, experts increasingly rely on scientific and experimental methods to select the most appropriate option from multiple alternatives. Among these, Multi-Criteria Decision Making (MCDM) techniques hold significant importance and are widely used across various fields (Alvarez et al., 2021). MCDM techniques help in ranking, prioritizing, or identifying optimal solutions based on predefined decision criteria, their relative importance, and potential costs and benefits (Odu, 2019). The selection of an MCDM technique depends on the specific context being analyzed (Tzeng and Huang, 2011), with several techniques available, such as AHP, BWM, DEMATEL, and ISM. The Analytic Hierarchy Process (AHP) is commonly used in MCDM but has drawbacks like redundant pairwise comparisons and consistency issues. To address these, the Best-Worst Method (BWM) was developed, offering an alternative for decision-making processes (Mi et al., 2019). BWM has gained popularity in various fields, including studies on human factors and safety. For instance, Mirzaei Aliabadi et al. (2022) applied BWM to assess human errors in the mining industry. While Trivedi et al. (2024) used a BWM-TOPSIS hybrid model for road safety evaluation. Interpretive Structural Modeling (ISM) is another technique used to transform ambiguous mental models into clear and observable models. ISM breaks down complex systems into components, builds a multi-level structural model, and utilizes insights from challenging scenarios to develop solutions (Sorooshian et al., 2023). ISM has proven efficient and versatile, being applied in various studies. For example, Shakerian et al. (2019) used it to model unsafe behaviors and Liu et al. (2018) applied it in safety management for subway construction. Fuzzy Cognitive Maps (FCMs) provide a graph-based method for knowledge representation, illustrating a set of concepts in a specific domain through cause-and-effect relationships (Felix et al., 2019). FCMs have also been extensively applied in safety-related research. For instance, Azadeh et al. (2014) used FCMs to assess resilience engineering factors in a petrochemical plant. While Dong et al. (2022) employed FCMs for aviation safety risk assessments.

This research expands on prior studies offering a comprehensive analysis of factors influencing team cognition within ERTs. Because of the crucial function of these teams during crises, investigating the cognitive factors affecting their efficacy is paramount. Examining team cognition in emergency contexts and identifying key influencing factors among team members provides valuable insights for managers to enhance team performance. Managers can improve team efficiency by understanding their shared cognition in emergencies and using that knowledge to develop effective strategies. This study aims to identify TCSFs in ERTs using the meta-synthesis method, weight these factors through the BWM, determine the influence of TCSFs on team cognition performance using ISM, and model the relationships between TCSFs with FCM.

## 2 Methods

### 2.1 Study design

**Figure 1:**
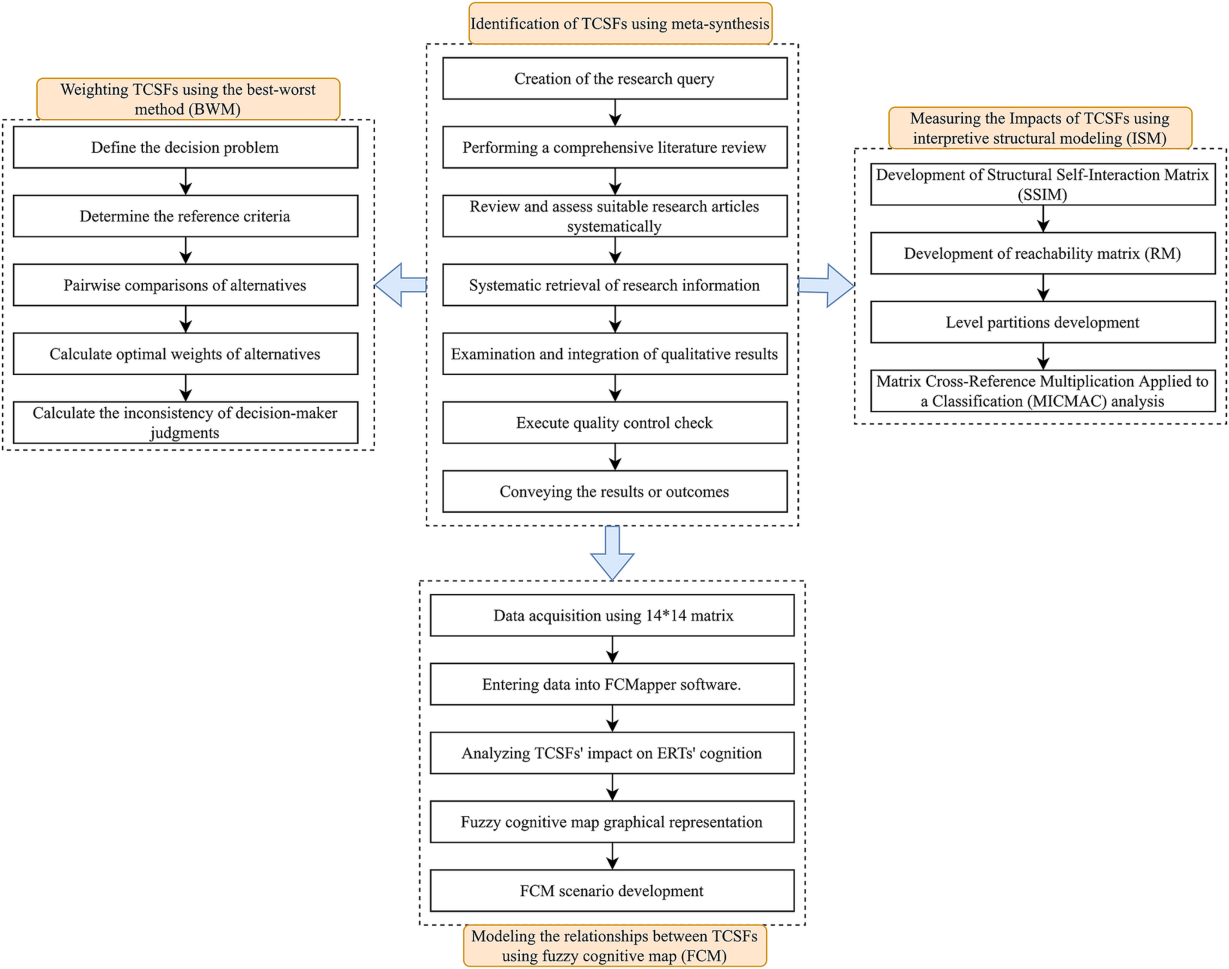
Outlining diagram of the study process.

The present investigation is a cross-sectional analytical study with the objective of discerning the team cognition factors influencing response teams in emergency situations. The study further aims to assign weights to these factors and construct models using multi-criteria decision-making techniques. Meta-synthesis features were employed in this study to identify Team Cognition Shaping Factors (TCSFs). Additionally, three techniques—BWM, ISM, and FCM—were utilized to model and prioritize these identified factors. The study process is illustrated in Figure 1.

### 2.2 Expert panel

Multi-criteria decision making methods endorse the incorporation of expert opinions, employing management techniques like brainstorming and the nominal group technique. This contributes to the establishment and weighting of contextual relationships among variables. To accomplish this, it is crucial to involve experts from both industry and academia. These experts should possess a thorough understanding of the problem at hand to adeptly identify and articulate the nature of contextual relationships among the factors. During this study, the initial phase included the selection of expert panel members who were actively engaged and proficient in the field of emergency management. The chain sampling approach was employed for this selection process, ultimately identifying 17 experts (Table 1). The selection criteria for the experts included:

- Possession of a PhD in Occupational Health and Safety, Ergonomics, Industrial-Organizational Psychology, or Industrial Safety.
- A Bachelor’s or Master’s degree in Occupational Health Engineering, Ergonomics, Industrial Safety, HSE, Emergency Management, or rescue and relief management with at least 5 years of professional experience in the mining or related industries.
- Expertise in the area of ERTs, team cognition, or organizational safety through academic publications or significant practical experience.

**Table 1:** Expert panel characteristics.

Expert ID	Field of expertise	Degree of education	Work experience (years)
E1	Head of firefighting unit	BSc in industrial and occupational safety	18
E2	Expert of firefighting unit	BSc in industrial and occupational safety	8
E3	Expert of firefighting unit	BSc in health, safety and environment engineering	8
E4	HSE expert	MSc in health, safety and environment engineering	15
E5	HSE expert	MSc in Occupational health and safety	12
E6	HSE expert	BSc in health, safety and environment engineering	8
E7	HSE expert	BSc in health, safety and environment engineering	10
E8	HSE expert	BSc in safety engineering	10
E9	Head of Relief and Rescue Unit	BSc in in Relief and Rescue	12
E10	Expert of Relief and Rescue Unit	BSc in in Relief and Rescue	12
E11	Expert of Relief and Rescue Unit	BSc in in Relief and Rescue	8
E12	Head of emergency management unit	MSc in emergency management	21
E13	Expert of emergency management unit	BSc in emergency management	12
E14, E15, E16, E17	HSE Expert	PhD in occupational health and safety	15, 17, 16, 12

The experts participated in multiple rounds of consultation. Structured questionnaires were employed to capture their insights systematically, and iterative feedback was gathered to refine the contextual relationships among variables. To reduce bias and improve input reliability, the consistency of judgments was evaluated using the kappa coefficient and consistency ratio using BWM. Also, the study used a multidisciplinary expert panel to produce a thorough and robust evaluation of the factors affecting team cognition success in ERTs (Farhadi et al., 2023).

All three analysis techniques (BWM, ISM, and FCM) used the opinions of all 17 study experts. The evaluation process was maintained using a comprehensive approach. This involved a single expert panel contributing to the weighting of factors (BWM), the hierarchical structuring (ISM), and the causal relationship modeling (FCM). Employing a unified expert panel across all methods enhanced the findings’ robustness through the maintenance of a consistent knowledge base and the reduction of variability in expert opinions.

### 2.3 Identification of TCSFs based on meta-synthesis approach

The current study employed the meta-synthesis approach as a qualitative method to identify factors influencing team cognition within Emergency Response Teams (ERTs). This approach provides a deeper understanding of the phenomenon by synthesizing qualitative studies, thus complementing systematic reviews and meta-analyses (Atkins et al., 2008). Unlike quantitative methods that focus on statistical data, meta-synthesis takes an exploratory stance, integrating interrelated qualitative studies to generate novel insights (Mohammed et al., 2016). It involves re-conceptualization and interpretation rather than merely summarizing findings, maintaining the original meaning while avoiding oversimplification (Walsh and Downe, 2005). The outcomes of this synthesis can lead to new theories, conceptual models, identification of research gaps, and a broader understanding of existing knowledge (Shakerian et al., 2019). The meta-synthesis approach follows several key steps, which are outlined next.

#### 2.3.1 Step 1: research query development

Formulating a precise research question is a vital step in the meta-synthesis process, helping researchers establish criteria based on the research domain and relevant variables (Hoon, 2013). This study explored existing literature on team cognition to identify specific problems or phenomena. A well-defined conceptual framework was established, considering guiding theories or competing models. Due to the limited research on team cognition in emergency response, the study aims to introduce new concepts in this field. The central research question guiding this inquiry is: “What factors exert influence on team cognition within response teams during emergency situations?”

#### 2.3.2 Step 2: performing a comprehensive literature review

To create a comprehensive list of studies for qualitative meta-synthesis, researchers must undertake a meticulous process (Tong et al., 2012). After identifying relevant keywords, the current study systematically reviewed published articles across several databases, including PubMed, Web of Science, Scopus, Google Scholar, SID, and ISC, from 1976 to 2023. A cross-referencing strategy was also employed to trace articles through their references, ensuring comprehensive access to relevant literature. Non-English language articles, conference presentations, unpublished works, correspondence, recommendations, animal studies, and case reports were excluded. The search focused on terms related to “team cognition” and “emergency response management,” using four keywords: Emergency, Management, Teams, and Cognition, as outlined in Table 2.

**Table 2:** Final search query.

Variable	Search query
Cognition	“cogniti*” OR “information process*” OR “information shar*” OR “mental model*” OR “sensemak*” OR “sense-mak*” OR “situation* awareness” OR “transactive memory system*” OR “Strategic consensus” OR “metacogniti*” OR “decision making” OR “perception*” OR “interpretation*” OR “mental process*” OR “macrocogniti*” OR “recogniti*”
Management	“command*” OR “collaborat*” OR “coordinat*” OR “interacti*” OR “manag*” OR “mitigat*” OR “operat*” OR “plan*” OR “prepar*” OR “recover*” OR “respon*”
Emergency	“emergenc*” OR “cris*” OR “disaster*” OR “incident*” OR “catastrophe”
Team	“interteam*” OR “inter team*” OR “multiteam*” OR “multi-team” OR “team*”

After establishing the search terms, the search was conducted for each database, and duplicate articles were removed. The following eligibility criteria were applied to select articles for inclusion in the meta-synthesis:

- The study must evaluate emergencies and crises related to industrial settings, excluding less relevant crises such as cyber crises, terrorist attacks, and various healthcare-related crises.
- The focus must be on teams, excluding studies on individuals, computing systems, or infrastructures.
- Studies with limited relevance to team cognition, such as those addressing team moral atmosphere, cognitive empathy, and leader emotions, were also excluded.

#### 2.3.3 Step 3: review and assess suitable research articles systematically

At this stage, the emphasis was on evaluating eligible studies and identifying key characteristics for assessment. This process includes developing a tool to compare studies based on criteria such as study objectives, data collection methods, data analysis techniques, and findings. A ranking system is used to facilitate the comparison of qualified information for the synthesis process.

A typology of conclusions by Sandelowski and Barroso (2003) was used to categorize collected data based on the study’s objectives and content, aiding in the selection of studies for the meta-synthesis. The Critical Appraisal Skills Program (CASP) assessed research quality through 10 questions focusing on accuracy, validity, and significance. Rigor questions evaluate the appropriateness of research methods, Credibility assesses the clarity of findings, and Relevance measures their usefulness. Each paper received a total score, with articles scoring below 30 excluded from analysis based on a 50-point Rubric scale. CASP scores were categorized as follows: 41–50 (excellent), 31–40 (very good), 21–30 (good), 11–20 (medium), and 0–10 (weak) (Finfgeld-Connett, 2018).

In our study, a total of 8,866 resources were initially identified, among which 8,776 studies were excluded due to ineligibility based on journal information, title, abstract, and content. Ultimately, 87 studies were retained for further analysis.

#### 2.3.4 Step 4: systematic retrieval of research information

The conclusive results extracted from the articles were categorized based on their characteristics, including paper title, author, year of publication, etc., and the research methodology employed. In the case of the final selected articles, information pertaining to the TCSFs within ERTs was extracted using a pre-designed table.

#### 2.3.5 Step 5: examination and integration of qualitative results

During this step, the outcomes derived from the qualitative meta-synthesis process were presented. A well-crafted illustration of findings is essential to assist various audiences in applying the meta-synthesis and transitioning from research to practical applications.

#### 2.3.6 Step 6: execute quality control check

In the meta-synthesis methodology, researchers utilize the Kappa coefficient to assess study quality. This non-parametric statistical method measures agreement levels between different scales or evaluators, producing a value between −1 and +1, where a value closer to +1 indicates higher agreement, while a value near −1 suggests low agreement. In this study, the Kappa index was calculated by comparing categories from the researcher and an expert, focusing on similarities and differences to ensure reliability (Shakerian et al., 2019). The agreement percentage was 92.8%, with Cohen’s *K* index value of 0.63, indicating substantial agreement between the two raters.

#### 2.3.7 Step 7: conveying the results or outcomes

In this step, the research findings were presented. A total of 87 papers were analyzed, and their main results were extracted according to the study’s objectives. These findings were categorized into 13 dimensions, named by the researcher following the meta-synthesis methodology (Sandelowski et al., 1997). To highlight the importance of these dimensions and explore potential hierarchical relationships among the factors, the BWM, ISM, and FCM methodologies were applied after extracting all the TCSFs.

### 2.4 Weighting factors affecting team cognitive performance using the BWM

The BWM, introduced by Rezaei (2015), is a compensatory Multi-Attribute Decision Making (MADM) technique that involves pairwise comparisons of the best and worst criteria or alternatives against others. When a decision maker identifies N evaluation criteria for the MADM problem, a pairwise comparison is performed to prioritize and rank the criteria relative to one another. This process is supported by constructing a pairwise comparison matrix as follows (Alinezhad and Khalili, 2019):


$$P=\begin{array}{c} c_{1}\\ \vdots \\ c_{n} \end{array}\;\begin{array}{c} c_{1}\;\ldots \;c_{n}\\ \left[\begin{array}{ccc} P_{\left(1,1\right)} & \cdots & P_{\left(1,n\right)}\\ \vdots & \ddots & \vdots \\ P_{\left(n,1\right)} & \cdots & P_{\left(n,n\right)} \end{array}\right] \end{array}$$

Where P is Pairwise comparison matrix of identified criteria, $P_{\left(i,j\right)}$ is pairwise comparison of the i-th criterion with the j-th criterion.

In the BWM, decision makers can use any numerical scale for pairwise comparisons, but a 1 to 9 scale is recommended (Table 3). This scale is commonly used to compare criteria or alternatives effectively.

**Table 3:** The 1–9 scale for pairwise comparison in BWM method.

Decision maker preference intensity	Definition
1	Equal importance
3	The average preference of the i-th criterion over the j-th criterion
5	Strong preference of i-th criterion over j-th criterion
7	Very strong preference of the i-th criterion over the j-th criterion
9	The i-th criterion is highly preferred over the j-th criterion
2, 4, 6, 8	Intermediate values between two adjacent judgments

The BWM was chosen for this study because of its efficiency and reliability in multi-criteria decision-making contexts. In contrast to traditional methods like AHP, BWM uses fewer comparisons, reducing the cognitive load on experts without compromising accuracy. Furthermore, BWM enhances judgment consistency by using a consistency ratio to check expert reliability, thus guaranteeing dependable results. This is advantageous in ERTs. By effectively using expert knowledge, BWM prioritizes TCSFs, enabling strategic resource allocation and better team performance in high-stakes situations (Rezaei, 2016).

The following section provides a step-by-step explanation of the BWM technique (Rezaei, 2015):

#### 2.4.1 Step 1: define the decision problem

Step 1 of the BWM involves establishing a framework for the decision-making process. The accuracy of this step is crucial, as it significantly influences the coherence of the final outcome. The key objective is for the decision-maker to mathematically define the decision problem using a set of criteria. A Decision Matrix is typically used for this purpose, where the decision-maker evaluates alternatives based on each criterion and then normalizes the results for each column [ $r_{\left(i,j\right)}$ ]. The final Decision Matrix possesses the following characteristics:


$$\overset{m}{\underset{i=1}{\sum }}r_{\left(i,j\right)}=1\forall j$$

Based on this, the decision problem in this study was defined as identifying and prioritizing the key factors influencing team cognition in ERTs.

#### 2.4.2 Step 2: determine the reference criteria

In the BWM, each element in the pairwise comparison matrix is used as either a reference or secondary comparison. Reference comparisons are the primary focus, where the decision-maker evaluates specific criteria to identify the most important (best) and least important (worst) criteria. This involves a comprehensive and subjective assessment of all predefined criteria to determine their significance. Depending on the decision-maker’s cognitive reasoning, more than one reference criterion (best or worst) can be selected for further evaluation (Rezaei, 2015).

In this stage, each expert independently selected the best and worst criteria among the factors influencing team cognition, which were identified through the meta-synthesis process, based on their professional expertise and judgment.

#### 2.4.3 Step 3: pairwise comparisons of alternatives

The BWM relies on reference comparisons, where the best criterion is compared with all others, and the remaining criteria are compared with the worst criterion. A numerical scale of 1–9 is recommended for these comparisons. These comparisons generate two vectors: one for best to others (AB) and another for others to worst (AW). These vectors are essential for ranking and evaluating criteria in decision-making (Rezaei, 2015).

During this stage, each factor was assessed through pairwise comparisons against the best and worst criteria identified in the prior step, allowing experts to determine its relative significance in shaping team cognition.

#### 2.4.4 Step 4: calculate optimal weights of alternatives

In this step, the information collected earlier is amalgamated to assign suitable weights to each criterion. The following equations can be used to calculate the weight of criterion j, assuming that each pairwise comparison results in the division of weights among the compared criteria (Rezaei, 2015):


PB.j=wBwj∀j

Pj.W=wjww∀j

Where $w_{i}$ , $w_{j}$ , $w_{B}$ and $w_{w}$ are weights allocated sequentially to criteria i, j, best, and worst, respectively (Rezaei, 2015).

#### 2.4.5 Step 5: calculate the inconsistency of decision-maker judgments

The MADM techniques that rely on pairwise comparisons can be vulnerable to inconsistencies due to errors in judgment by decision-makers. Therefore, the final step in the decision-making process should involve assessing the logical consistency of these evaluations. In BWM, which also uses pairwise comparisons, Rezaei (2015) introduced a method for measuring inconsistencies specific to BWM’s structure. A lower inconsistency ratio indicates a higher degree of consistency in the decision-making process. According to Rezaei study, inconsistency ratios below 0.1 are generally considered acceptable for deriving reliable conclusions (Rezaei, 2016).

Finally, all the aforementioned steps were implemented in Excel software, allowing for the calculation of the weights of the identified factors and the inconsistency ratio among expert opinions.

### 2.5 Measuring the impacts of TCSFs on team cognition performance of ERTs using ISM

The ISM developed by Warfield in 1973, is a technique used to analyze complex and subjective problems (Warfield, 1974). It is part of the causal mapping family and is particularly useful in multilevel research scenarios where predicting outcomes is challenging (Attri et al., 2013). ISM leverages expert insights to break down complex systems into manageable subsystems, creating a multilevel structural model (Agarwal et al., 2021). This method identifies and clarifies relationships among specific variables, allowing analysis of how one variable influences others (Khan et al., 2022). ISM method, embodies its name through its interpretive, structural, and modeling methodology. The interpretive aspect stems from the derivation of relationships among elements through the expressed opinions of a group of members. Structurally, ISM is rooted in an overall structure extracted from a complex set of variables. Lastly, the term “modeling” is aptly applied because specific relationships and the overall structure can be visually represented in a graphical model (Sushil, 2012). In the present study, ISM was employed to analyze the hierarchical relationship among TCSFs, as it is capable of modeling complex interdependencies in a systematic manner. ISM arranges TCSFs into levels according to their driving and dependence powers, thus giving a clear and actionable framework of their interrelationships (Kaur et al., 2023). By integrating expert judgments, ISM makes sure that the hierarchical model reflects practical insights, which is particularly important in the context of Emergency Response Teams. This structured approach enables organizations to identify the foundational factors driving higher-level outcomes, thus allowing strategic interventions aimed at enhancing team cognition and performance in high-stakes environments.

#### 2.5.1 Development of structural self-interaction matrix (SSIM)

In the ISM methodology, the analysis of factors involves establishing a contextual relationship characterized as either ‘leads to’ or ‘influences,’ indicating the impact one factor has on another. This relationship is determined using four symbols (Attri et al., 2013):

- “V” indicates that factor i influences factor j.
- “A” denotes that factor i is influenced by factor j.
- “X” signifies a bidirectional relationship, where factors i and j mutually influence each other.
- “O” represents no relationship, indicating that factors i and j are unrelated.

To construct the SSIM for the element under consideration, the group’s responses to each pairwise interaction between the factors were documented in a pairwise comparison matrix, including the TCSFs identified from the meta-synthesis stage.

#### 2.5.2 Development of reachability matrix (RM)

In the ISM approach, the next step is to create an initial reachability matrix from the SSIM by converting the SSIM into this matrix. The four symbols used in the SSIM are replaced with binary values (1 or 0) based on the following substitution rules (Sushil, 2012):

- If the (i, j) entry in the SSIM is V, then the (i, j) entry in the reachability matrix is set to 1, and the (j, i) entry is set to 0.
- If the (i, j) entry in the SSIM is A, then the (i, j) entry in the matrix is set to 0, and the (j, i) entry is set to 1.
- If the (i, j) entry in the SSIM is X, then the (i, j) entry in the matrix is set to 1, and the (j, i) entry is also set to 1.
- If the (i, j) entry in the SSIM is O, then the (i, j) entry in the matrix is set to 0, and the (j, i) entry is also set to 0.

In next step, Final reachability matrix is obtained through multiplicative relations, indicating if i leads to j and j leads to k, then i leads to k (1* in the initial matrix). Degree of dependence and influence are determined, representing impact on other goals and influence from other components, respectively. Influence is calculated from row sums, while dependence is obtained from column sums. The outcome provides component levels. Components with significant dependencies are positioned at the top, and those with high influence are at the bottom based on the final reachability matrix (Sushil, 2012).

#### 2.5.3 Level partitions development

The final reachability matrix is used to extract reachability and antecedent sets for each factor. The reachability set includes the factor and those it influences, while the antecedent set consists of the factor and those that influence it. By analyzing the intersection of these sets, factors are assigned levels. Factors with identical reachability and intersection sets are placed at the top level, indicating they do not influence others above them. Once a top-level factor is identified, it is excluded from further consideration, and the process repeats to determine the next levels. This continues until all factor levels are established, allowing for the construction of the digraph and the ISM model (Gardas et al., 2017).

#### 2.5.4 Matrix cross-reference multiplication applied to a classification (MICMAC) analysis

This method provides a visual representation of variables in structural-interpretive modeling based on their influence and dependence. A coordinate system is established, divided into four categories (Agarwal et al., 2021):

- Autonomous variables: these have weak dependence and influence, functioning independently with minimal impact on others.
- Dependent variables: these show weak influence but have a higher degree of dependence on other components.
- Linkage variables: characterized by strong influence and dependence, they are dynamic and can affect other components while being influenced by them as well.
- Independent variables: these possess strong influence but weak dependence, serving as pivotal components that can impact the rest of the variables.

All the steps mentioned above for ISM were implemented using Excel software, through which the TCSFs were ranked, and the influence levels among them were determined.

### 2.6 Modeling the relationships among TCSFs using FCM

Kosko (1986) introduced FCMs in 1986 as knowledge-based recurrent neural networks for modeling and simulating dynamic systems. With this approach, data can be evaluated using directed graphs and connection matrices. As a result, FCMs represent fuzzy graph structures for illustrating causal reasoning frameworks. Graph structures like these facilitate the representation of causal relationships among different factors, especially supporting recursive chaining between them. Such structures provide the possibility of expanding knowledge bases by connecting different FCMs (Papageorgiou and Salmeron, 2013). FCMs are well-suited for unsupervised data and rely on expert opinions, depicting the world as classes with interconnected relationships. Its feedback enables experts to depict causal relationships in their problems freely and deduce causal links from basic data. Considering FCM as a dynamical system, its equilibrium behavior is viewed as forward-evolved inference (Papageorgiou, 2012). FCMs employ a set of nodes to represent influential factors and utilize edges to illustrate the cause-and-effect relationships between these factors. If there are nodes labeled X1 to Xn in an FCM network, the edges eij connect these nodes with a fuzzy causal interval of [−1, 1]. The matrix E, defined as E = (eij), represents the weights of directed edges from Ci to Cj. E is referred to as the adjacency matrix of the FCM, also recognized as the connectivity matrix of the FCM. In this context, eij = 0 signifies no causality, eij > 0 implies a causal increase (Cj increases as Ci increases or Cj decreases as Ci decreases), and eij < 0 indicates a causal decrease or negative causality (Cj decreases as Ci increases, or Cj increases as Ci decreases) (Felix et al., 2019). The synergy of profound knowledge, valuable experience, and meticulous scripting is imperative for designing a desired Fuzzy Cognitive Map (FCM) and achieving optimal results. A comprehensive examination of the assessed system, formulation of cause-and-effect variables, and collaboration with an expert team are equally crucial (Bakhtavar and Shirvand, 2019). In the current study, a 14×14 matrix was constructed, comprising the 13 factors identified during the meta-synthesis phase, along with the team cognition factor (labeled as X14). This matrix served as the foundation for building the Fuzzy Cognitive Map (FCM), which incorporated insights gathered from the expert panel. At this stage, knowledge integration on all 17 panel members about finding cause-effect relationships among factors takes place. Further, the experts map every paired factor in their fuzzy casual interval that, when summed, forms the adjacency matrix. Thus, this adjacency matrix represents causal direct and indirect influences between the factors while at the same time reflecting consistencies and insights regarding their profundity in knowledge provided by these experts. The same reason, that it can model complex dynamic systems, FCM is utilized here in modeling the causes of TCSFs. The cause-effect relationship among the factors will be captured through the inclusion of uncertainty and ambiguity, which has been implemented by implementing fuzzy logic (Yusuf et al., 2022). This again is very valid in the case of ERTs, where every decision-making element is interrelated and uncertain in nature. FCMs also enable scenario-based simulations that examine the effects of interventions on team cognition. By integrating expert judgment and providing quantitative results regarding strength and direction of relationships, FCMs offer a sound framework for analyzing and optimizing team cognition in high-stakes environments.

#### 2.6.1 Scenario development

Scenario development involves identifying the key factors that can bring about the most significant change in the phenomenon under investigation, such as team cognition in our study. This process consists of three main steps (Zanjirchi et al., 2020; Bevilacqua et al., 2018):

1. Identification of most significant factors: in this step, the most influential factor is determined by calculating its total effect value. By analyzing the total effect values, the most critical concepts are ranked accordingly.
2. Assessment of the impact of scenario implementation: to assess which factor has the greatest influence on team cognition, scenarios are modified based on the crucial factor identified in the previous step. The FCM Scenarios module of the FCMapper software is utilized for this purpose. Comparing the outcomes of different scenarios reveals the effectiveness of each scenario.
3. Selection of the best path: in this phase, the principal pathways affecting team cognition are deduced to identify the key concept sequences. These pathways are determined based on the results of the preceding step. This enables industrial managers to delineate appropriate actions for enhancing team cognition.

The current study would present an all-integrative approach to view TCSFs with BWM, ISM, and FCM. In the integrated framework, the BWM gives priority of TCSFs on the basis of expert judgment and further clarifies their hierarchical order with ISM. The result was further used for a FCM model to explore scenarios by finding the flow that occurs when the values of the elements are changed within the system. This multi-method approach integrates static prioritization, structural modeling, and dynamic analysis in providing actionable insights that enhance team cognition and performance in ERTs. The current study makes an integrative analysis of the quantification of importance done by BWM, the hierarchical relationships developed by ISM, and dynamic scenario simulations developed by FCM to present a comprehensive, robust, and unbiased analysis of factors influencing team cognition in ERTs.

### 2.7 Software

The entire process of calculating the BWM and ISM steps was executed using custom Excel software developed by the researchers involved in this study. Additionally, the relationships were analyzed and the FCMs were constructed using the FCMapper software, which operates within the Excel environment. Furthermore, Pajek software was employed for examining the relationships and visualizing the FCMs.

## 3 Results

### 3.1 Meta-synthesis analysis

After conducting an in-depth review of the selected articles and evaluating them based on the CASP, a total of 87 studies were deemed eligible for inclusion. Consequently, the team identified key factors shaping team cognition within ERTs (see Supplementary Table 1). these crucial sub-factors can be categorized into TCSFs, including: “Team maturity (The team members harmonization),” “Inefficient 4Cs (communication, coordination, cooperation and collaboration),” “Using technology and tools,” “Failure in decision-making,” “Improper team training programs,” “The quality of information and information sharing process,” “Team members Incorrect sensemaking of the crisis,” “Lack of procedures or incomplete procedures of teamwork,” “Team cultural, contextual, organizational and social conditions,” “Actual situation knowledge (or awareness) and perceived situation knowledge (or awareness),” “Leadership behavior and performance,” “prior knowledge, mental models and transactive memory system,” “Monitoring the performance of the emergency response team”.

### 3.2 Weighting and ranking of TCSFs using BWM

The study’s findings, based on BWM analysis, identify “Team maturity (The team members harmonization)” and “Inefficient 4Cs (communication, coordination, cooperation, and collaboration)” as the most influential factors in shaping team cognition within Emergency Response Teams (ERTs), with weights of 0.132 and 0.112, respectively (Table 4). These insights highlight the importance of cohesive team dynamics and the detrimental effects of poor 4Cs on team cognition. Conversely, factors such as “Team members’ Incorrect sensemaking of the crisis” and “Prior knowledge, mental models, and transactive memory system” were found to have minimal influence, with weights of 0.046 and 0.050, respectively. These lower-impact factors suggest areas where further intervention could enhance team performance. The BWM Inconsistency Ratio of 0.067 indicates a high consistency in the pairwise comparison judgments used for the analysis.

**Table 4:** Team cognition shaping factors (TCSFs) weight and rank based on BWM.

New TCSFs	Symbol	Number of articles related to each factor	Rank	BWM Weight	BWM Rank
Team maturity (the team members harmonization)	X1	50	1	0.132399	1
Inefficient 4Cs (communication, coordination, cooperation and collaboration)	X2	41	2	0.112265	2
Using technology and tools	X3	25	7	0.053967	11
Failure in decision-making	X4	30	4	0.073422	6
Improper team training programs	X5	29	5	0.090662	4
The quality of information and information sharing process	X6	40	3	0.06013	9
Team members incorrect sense making of the crisis	X7	9	12	0.046225	13
Lack of procedures or incomplete procedures of teamwork	X8	15	9	0.074164	5
Team cultural, contextual, organizational and social conditions	X9	27	6	0.057035	10
Actual situation knowledge (or awareness) and perceived situation knowledge (or awareness)	X10	24	8	0.073077	7
Leadership behavior and performance	X11	15	9	0.111343	3
Prior knowledge, mental models and transactive memory system	X12	13	11	0.050876	12
Monitoring the performance of the emergency response team	X13	8	13	0.064434	8

### 3.3 TCSFs impacts modeling in ERTs using ISM

#### 3.3.1 Structural self-interaction matrix (SSIM) of TCSFs

The SSIM presented in Table 5 is derived from the expert panel’s pairwise assessments of criteria, reflecting their collective insights into the various factors under consideration.

**Table 5:** Structural self-interaction matrix.

	X1	X2	X3	X4	X5	X6	X7	X8	X9	X10	X11	X12	X13
X1	−	2	−1	2	−1	2	2	−1	−1	−1	−1	−1	−1
X2	2	−	−1	2	−1	1	2	−1	−1	−1	−1	−1	−1
X3	1	1	−	1	0	1	1	0	−1	1	1	1	1
X4	2	2	−1	−	−1	−1	−1	−1	−1	−1	−1	−1	−1
X5	1	1	0	1	−	1	1	0	1	1	1	1	0
X6	2	−1	−1	1	−1	−	2	−1	−1	−1	−1	2	−1
X7	2	2	−1	1	−1	2	−	−1	2	−1	−1	−1	−1
X8	1	1	0	1	0	1	1	−	−1	1	1	1	1
X9	1	1	1	1	−1	1	2	1	−	1	1	1	1
X10	1	1	−1	1	−1	1	1	−1	−1	–	−1	2	−1
X11	1	1	−1	1	−1	1	1	−1	−1	1	–	1	0
X12	1	1	−1	1	−1	2	1	−1	−1	2	−1	−	−1
X13	1	1	−1	1	0	1	1	−1	−1	1	0	1	–

#### 3.3.2 Initial and final reachability matrix (RM) of TCSFs

In this phase, the creation of a reachability matrix unfolds, initiated by utilizing the Structural Self-Interaction Matrix (SSIM). Subsequently, a conversion to a binary matrix takes place, where values 1, −1, 2, and 0 are substituted with 0s and 1s following specific rules: Within each row, numbers 1 and 2 are replaced with 1, while numbers −1 and 0 are substituted with 0. The resultant binary matrix is elegantly presented in Table 6.

**Table 6:** Initial reachability matrix.

	X1	X2	X3	X4	X5	X6	X7	X8	X9	X10	X11	X12	X13
X1	1	1	0	1	0	1	1	0	0	0	0	0	0
X2	1	1	0	1	0	1	1	0	0	0	0	0	0
X3	1	1	1	1	0	1	1	0	0	1	1	1	1
X4	1	1	0	1	0	0	0	0	0	0	0	0	0
X5	1	1	0	1	1	1	1	0	1	1	1	1	0
X6	1	0	0	1	0	1	1	0	0	0	0	1	0
X7	1	1	0	1	0	1	1	0	1	0	0	0	0
X8	1	1	0	1	0	1	1	1	0	1	1	1	1
X9	1	1	1	1	0	1	1	1	1	1	1	1	1
X10	1	1	0	1	0	1	1	0	0	1	0	1	0
X11	1	1	0	1	0	1	1	0	0	1	1	1	0
X12	1	1	0	1	0	1	1	0	0	1	0	1	0
X13	1	1	0	1	0	1	1	0	0	1	0	1	1

By employing the ISM approach, the final RM was derived from the initial RM. This matrix elucidates the driving forces and interdependencies among each TCSF. Leveraging this matrix, the factors were categorized into two hierarchical levels, as illustrated in Table 7.

**Table 7:** Final reachability matrix and the driving/dependence power of factors.

	X1	X2	X3	X4	X5	X6	X7	X8	X9	X10	X11	X12	X13
X1	1	1	0	1	0	1	1	0	1*	0	0	1*	0
X2	1	1	0	1	0	1	1	0	1*	0	0	1*	0
X3	1	1	1	1	0	1	1	0	1*	1	1	1	1
X4	1	1	0	1	0	1*	1*	0	0	0	0	0	0
X5	1	1	1*	1	1	1	1	1*	1	1	1	1	1*
X6	1	1*	0	1	0	1	1	0	1*	1*	0	1	0
X7	1	1	1*	1	0	1	1	1*	1	1*	1*	1*	1*
X8	1	1	0	1	0	1	1	1	1*	1	1	1	1
X9	1	1	1	1	0	1	1	1	1	1	1	1	1
X10	1	1	0	1	0	1	1	0	1*	1	0	1	0
X11	1	1	0	1	0	1	1	0	1*	1	1	1	0
X12	1	1	0	1	0	1	1	0	1*	1	0	1	0
X13	1	1	0	1	0	1	1	0	1*	1	0	1	1

*Transitivity is incorporated into the final reachability matrix values.

#### 3.3.3 Level partitions of TCSFs

In conclusion, a hierarchical model was developed to illustrate the hierarchy of TCSFs influencing team cognition among ERT members. The process involved partitioning the reachability matrix based on reachability and antecedent sets for each variable through iterative steps. The TCSFs were categorized into five levels denoted as L1, L2, L3, L4, and L5, as detailed in Table 8.

- L1 represents the direct cause layer and comprises five factors: “Team maturity (The team members harmonization),” “Inefficient 4Cs (communication, coordination, cooperation, and collaboration),” “Failure in decision-making,” “The quality of information and information sharing process,” and “Team members’ Incorrect sensemaking of the crisis.”
- L2, L3, and L4 represent important cause layers, encompassing seven factors: “Team cultural, contextual, organizational, and social conditions,” “Actual situation knowledge (or awareness) and perceived situation knowledge (or awareness),” “Prior knowledge, mental models, and transactive memory system,” “Leadership behavior and performance,” “Monitoring the performance of the emergency response team,” “Using technology and tools,” and “Lack of procedures or incomplete procedures of teamwork.”
- L5, the most crucial layer, consists of a single factor: “Improper team training programs.”

**Table 8:** Level partition-iterations of TCSFs.

Criteria	Reachability set (Ri)	Antecedent set (Si)	Intersection set (Ri ⋂ Si)	Level
X1	X1 X2 X4 X6 X7 X9 X12	X1 X2 X3 X4 X5 X6 X7 X8 X9 X10 X11 X12 X13	X1 X2 X4 X6 X7 X9 X12	L1
X2	X1 X2 X4 X6 X7 X9 X12	X1 X2 X3 X4 X5 X6 X7 X8 X9 X10 X11 X12 X13	X1 X2 X4 X6 X7 X9 X12	L1
X4	X1 X2 X4 X6 X7	X1 X2 X3 X4 X5 X6 X7 X8 X9 X10 X11 X12 X13	X1 X2 X4 X6 X7	L1
X6	X1 X2 X4 X6 X7 X9 X10 X12	X1 X2 X3 X4 X5 X6 X7 X8 X9 X10 X11 X12 X13	X1 X2 X4 X6 X7 X9 X10 X12	L1
X7	X1 X2 X3 X4 X6 X7 X8 X9 X10 X11 X12 X13	X1 X2 X3 X4 X5 X6 X7 X8 X9 X10 X11 X12 X13	X1 X2 X3 X4 X6 X7 X8 X9 X10 X11 X12 X13	L1
X9	X3 X8 X9 X10 X11 X12 X13	X3 X5 X8 X9 X10 X11 X12 X13	X3 X8 X9 X10 X11 X12 X13	L2
X10	X9 X10 X12	X3 X5 X8 X9 X10 X11 X12 X13	X9 X10 X12	L2
X12	X9 X10 X12	X3 X5 X8 X9 X10 X11 X12 X13	X9 X10 X12	L2
X11	X11	X3 X5 X8 X11	X11	L3
X13	X13	X3 X5 X8 X13	X13	L3
X3	X3	X3 X5	X3	L4
X8	X8	X5 X8	X8	L4
X5	X5	X5	X5	L5

#### 3.3.4 MICMAC analysis of TCSFs

**Figure 2:**
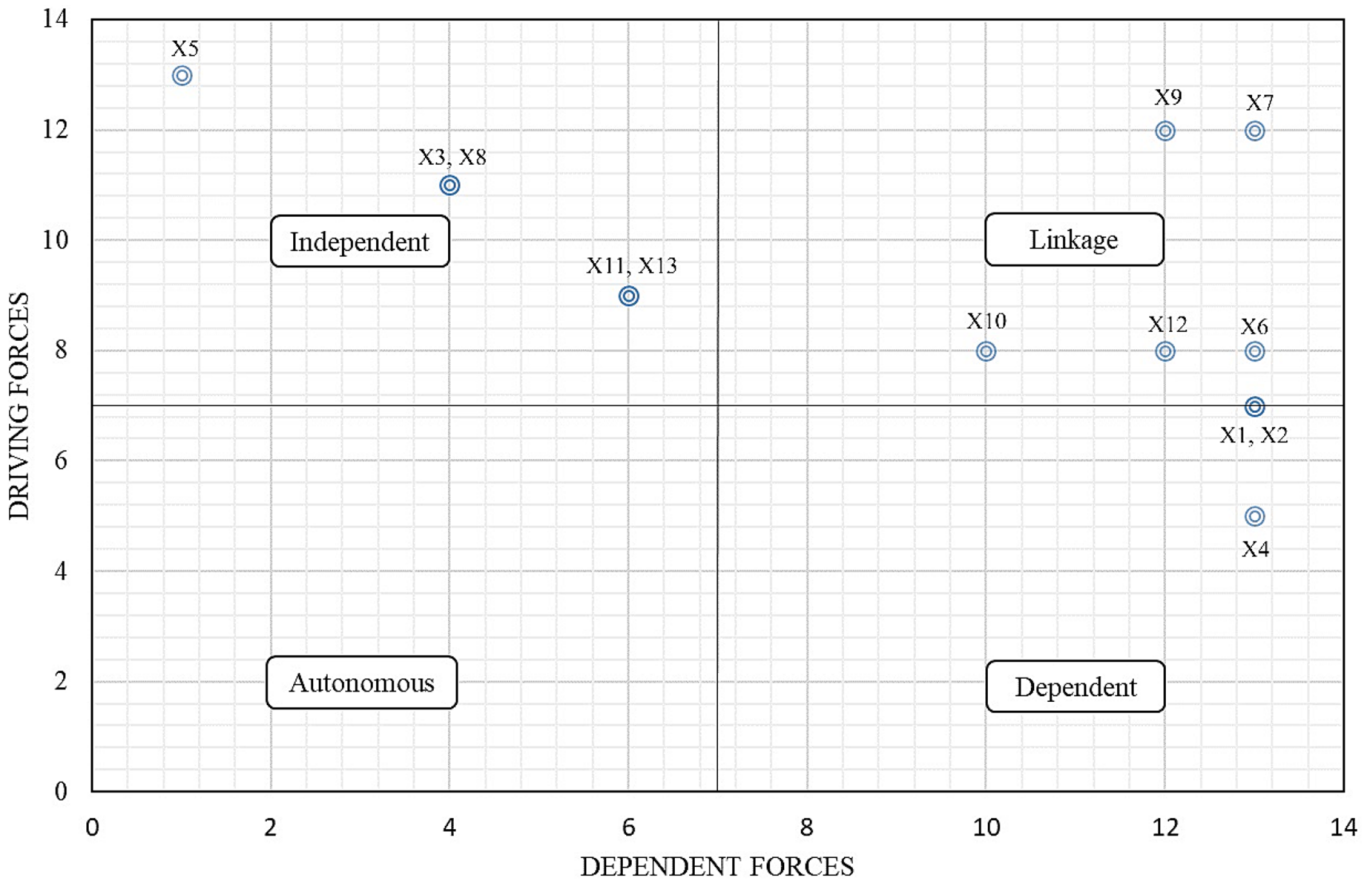
Matrix cross-reference multiplication applied to a classification (MICMAC) analysis to determine the status of TCSFs.

As depicted in Figure 2, the TCSFs “Improper team training programs,” “Using technology and tools,” “Lack of procedures or incomplete procedures of teamwork,” “Leadership behavior and performance,” and “Monitoring the performance of the emergency response team” are located in the independent cluster. This indicates that these factors are considered independent variables as they exert influence on other variables in the linkage cluster but are not significantly impacted by them. Understanding the positioning of TCSFs within these clusters provides valuable insights into their role and influence within the system being studied. It helps in identifying key drivers and understanding the dynamics of interactions among variables, thus informing decision-making processes aimed at improving overall performance and effectiveness.

### 3.4 TCSFs relationship modeling using FCM

Table 9, representing the final matrix of success obtained based on expert judgment, serves as crucial input data for the FCMapper software. This matrix encapsulates the relationships between TCSFs as perceived by domain experts. Through this modeling process, the FCMapper software facilitates the exploration of the complex dynamics inherent in the system under study. It allows for the identification of key drivers, the assessment of potential intervention points, and the evaluation of the overall impact of TCSFs on the desired outcome, which in this case is success.

**Table 9:** Fuzzy cognitive mapping (FCM) final matrix of success based on expert judgment.

	X1	X2	X3	X4	X5	X6	X7	X8	X9	X10	X11	X12	X13	X14
X1	0	0.83	0	−0.79	0	0.71	−0.72	0	0	0.64	0.61	0.48	0	0.82
X2	0.71	0	0	−0.73	0	0.83	−0.67	0	0	0.61	0.52	0.69	0	0.79
X3	0.35	0.71	0	0.49	0	0.71	−0.45	0	0	0.57	−0.33	0.58	0	0.59
X4	−0.59	−0.57	0	0	0	−0.55	0.48	0	0	−0.42	0.45	−0.41	0	−0.78
X5	−0.71	−0.72	−0.39	0.76	0	−0.69	0.52	0	0	−0.65	−0.66	0.57	0	−0.66
X6	0.69	0.73	0	−0.68	0	0	−0.67	0	0	0.69	0.58	0.66	0	0.7
X7	−0.64	−0.57	0	0.58	0	−0.54	0	0	0	−0.4	−0.65	0	0	−0.53
X8	−0.63	−0.59	−0.43	0.98	0	−0.57	0.45	0	0	−0.43	−0.66	−0.46	−0.61	−0.64
X9	0.73	0.59	0.46	−0.57	0.43	0.52	−0.49	0	0	0.52	0.56	0.54	0.51	0.65
X10	0.62	0.59	0	−0.59	0	0.72	−0.63	0	0	0	0.56	0.67	0	0.75
X11	0.85	0.73	0.58	−0.73	0	0.53	−0.52	0	0	0.52	0	0.45	0.61	0.69
X12	0.65	0.66	0	−0.49	0	0.53	−0.49	0	0	0.57	0.48	0	0	0.61
X13	0.51	0.6	0	−0.5	0	0.52	−0.31	0	0	0.33	0.48	0.34	0	0.57
Team cognition (X14)	0	0	0	0	0	0	0	0	0	0	0	0	0	0

**Figure 3:**
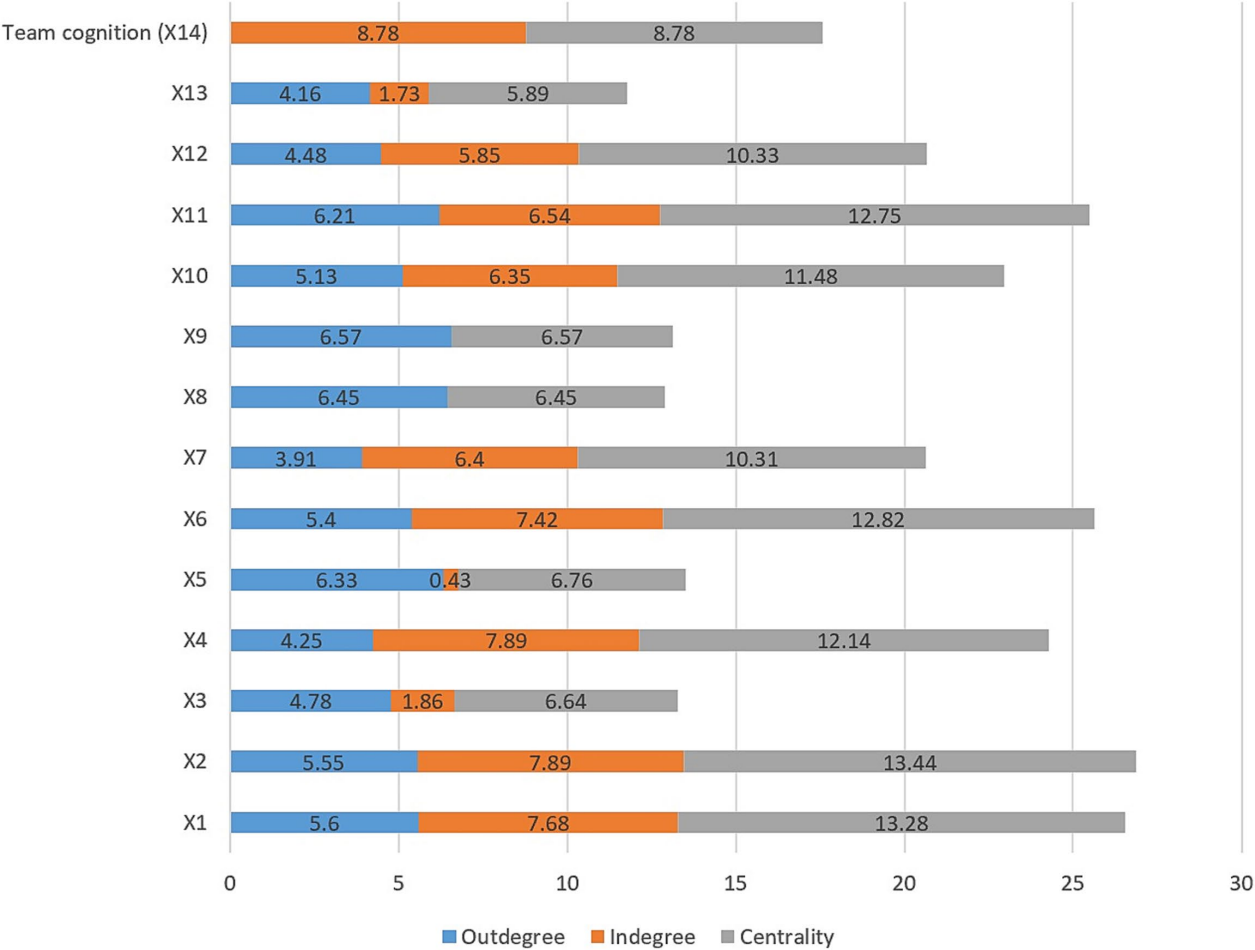
The relationships among TCSFs and the effect of them on team cognition of ERTs using the FCM.

The analysis in Figure 3, using FCMapper software, explores the relationships among team cognition shaping factors (TCSFs) through measures of outdegree, indegree, and centrality. Outdegree indicates each factor’s outgoing impact, while indegree shows the influence a factor receives. Centrality, calculated by summing indegree and outdegree, highlights the overall importance of each factor within the network. Factors X2 and X1 emerged as most impactful, with centrality values of 13.44 and 13.28, respectively, while X6, X11, and X4 also demonstrated strong influences, with centrality values of 12.82, 12.75, and 12.14, respectively. Team cognition (X14) held the highest indegree index at 8.78, underscoring its central role in TCSFs dynamics. These insights guide priority-setting for interventions, helping allocate resources to optimize team performance and achieve organizational objectives.

**Figure 4:**
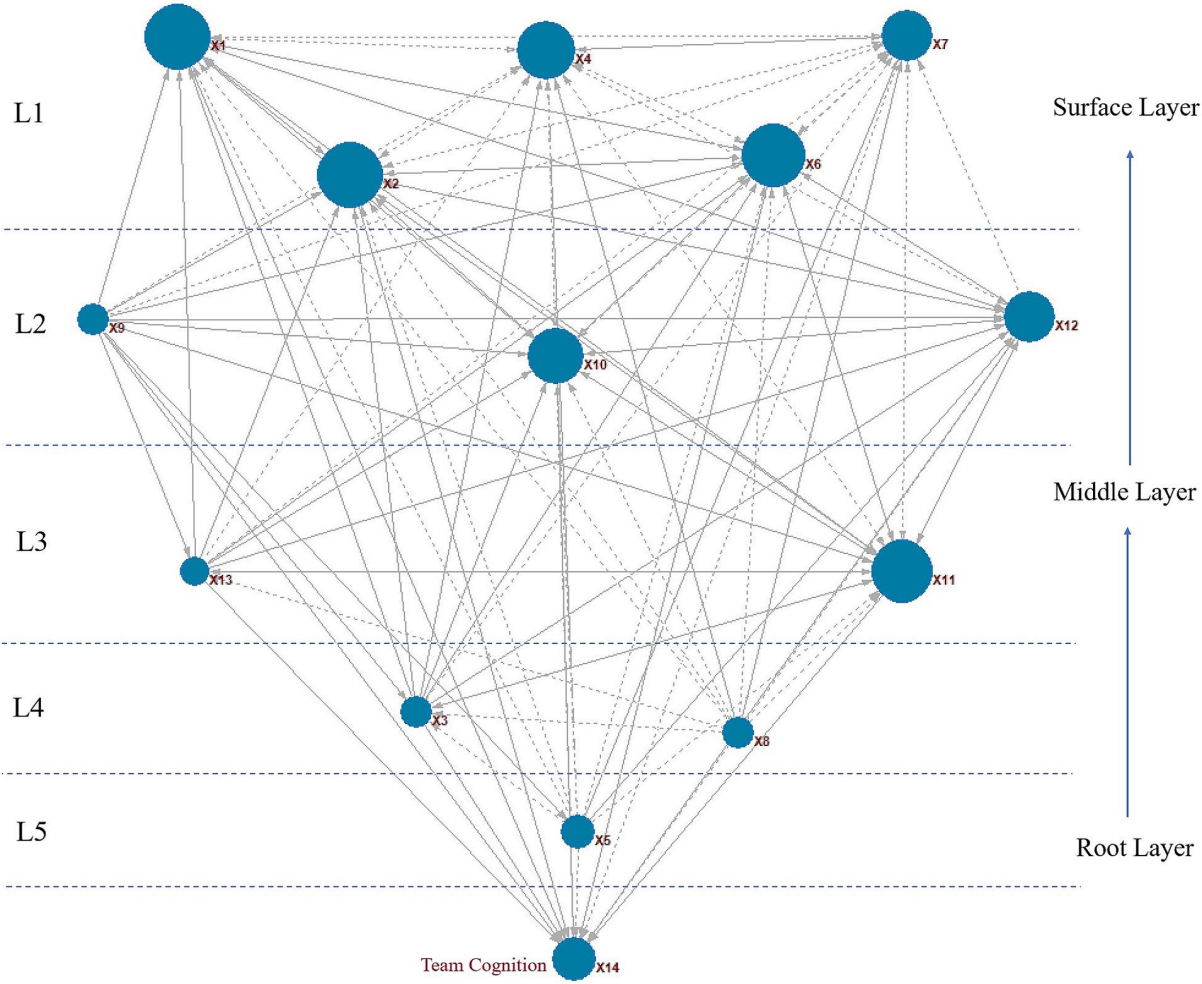
Diamond model of direct, indirect, and inverse relationships among TCSFs using FCM and ISM modeling.

Figure 4 displays a diamond model integrating ISM and FCM techniques to depict the hierarchical structure and interrelations among team cognition shaping factors (TCSFs). The diagram includes 116 connections, with 46 indicating negative effects (shown by black dashes) and positive effects represented by solid black lines. This visualization provides an in-depth view of dependencies within the TCSF system. Aligning with prior findings, factors X1 and X2 stand out for their substantial impact on the model, highlighting their crucial influence on system dynamics and reinforcing their importance in strategies aimed at enhancing team performance and achieving organizational goals.

### 3.5 FCM scenario analysis

#### 3.5.1 Identification of most significant factors

Based on the comprehensive analysis conducted across multiple stages, three factors have been identified for scenario development. These factors were selected based on their significance and impact within the ERTs system, as determined by various evaluation techniques:

- X2: This factor emerged with the highest weight in the evaluation using the BWM technique. Additionally, it exhibited the highest Centrality index score, signifying its critical importance within the network of TCSFs.
- X1: Similar to X2, X1 also attained the highest weight in the BWM evaluation and demonstrated a high centrality index score. Its prominence highlights its substantial influence on the system dynamics and outcomes.
- X5: This factor was identified as having the most importance according to the results of the ISM modeling. ISM revealed Factor X5 to be a pivotal element within the hierarchical structure of TCSFs, further emphasizing its significance in shaping the overall system behavior.

By selecting X1, X2, and X5 factors for scenario development, the aim is to explore potential interventions and strategies that leverage these key factors to enhance system performance and achieve desired outcomes. Scenario development involving these factors enables stakeholders to anticipate and prepare for various future scenarios, thereby fostering proactive decision-making and strategic planning.

#### 3.5.2 Assessment of the impact of scenario implementation

Table 10 FCM-scenario analysis reveals the effects of various Team Cognition Shaping Factors (TCSFs) across three scenarios:

1. Scenario 1 shows that “Improper team training programs” (X5) most strongly affects “Failure in decision-making” (X4) and “Leadership behavior and performance” (X11). Improving training programs could therefore significantly enhance decision-making and leadership within ERTs.
2. Scenario 2 highlights “Team maturity (The team members harmonization)” (X1) as a key influencer, impacting “Inefficient 4Cs” (X2), “Information quality and sharing” (X6), “Situation awareness” (X10), and “Leadership behavior” (X11). Strategies to improve team harmony, such as team-building, effective communication, and leadership training, may strengthen these areas.
3. Scenario 3 emphasizes the importance of addressing “Inefficient 4Cs” (X2), which most strongly influences “Failure in decision-making” (X4), “Incorrect sensemaking” (X7), and “Leadership behavior” (X11). Enhancing communication protocols, coordination, and cooperation among team members could mitigate these challenges and improve team cognition and performance in ERTs.

**Table 10:** The total effect of TCSFs and FCM-scenario results.

Concepts	Total effect	Scenario 1 (based on X5)				Scenario 2 (based on X1)				Scenario 3 (based on X2)			
		Results	changes	value	Definition	Results	changes	value	Definition	Results	changes	value	Definition
X1	0.990	0.994	0.004	3	Medium pos. change	0	−0.990	0	–	0.978	−0.011	6	High neg. change
X2	0.992	0.996	0.003	3	Medium pos. change	0.981	−0.011	6	High neg. change	0	−0.992	0	–
X3	0.736	0.800	0.063	2	High pos. Change	0.732	−0.004	7	Medium neg. change	0.733	−0.003	7	Medium neg. change
X4	0/043	0.024	−0.018	6	High neg. change	0.099	0.056	2	High pos. change	0.094	0.051	2	High pos. change
X5	0.734	0	−0.734	0	–	0.734	0.000	5	Very low pos. change	0.734	0.000	5	Very low pos. change
X6	0.990	0.994	0.004	3	Medium pos. change	0.978	−0.011	6	High neg. change	0.975	−0.014	6	High neg. change
X7	0.021	0.013	−0.007	7	Medium neg. change	0.046	0.025	2	High pos. change	0.044	0.023	2	High pos. change
X8	0.659	0.659	0.000	5	Very low pos. change	0.659	0.000	5	Very low pos. change	0.659	0.000	5	Very low pos. change
X9	0.659	0.659	0.000	5	Very low pos. change	0.659	0.000	5	Very low pos. change	0.659	0.000	5	Very low pos. change
X10	0.985	0.991	0.006	3	Medium pos. change	0.9704	−0.014	6	High neg. change	0.971	−0.014	6	High neg. change
X11	0.963	0.977	0.013	2	High pos. change	0.932	−0.031	6	High neg. change	0.937	−0.025	6	High neg. change
X12	0.993	0.990	−0.002	7	Medium neg. change	0.989	−0.004	7	Medium neg. change	0.986	−0.007	7	Medium neg. change
X13	0.787	0.789	0.001	3	Medium pos. change	0.783	−0.003	7	Medium neg. change	0.784	−0.003	7	Medium neg. change
Team cognition (X14)	0.996	0.998	0.001	3	Medium pos. change	0.991	−0.005	7	Medium neg. change	0.991	−0.005	7	Medium neg. change

#### 3.5.3 Selection of the best path

By focusing on concepts with a cumulative total effect greater than 0.98, Table 11 highlights the core elements that exert significant influence on the dynamics and outcomes of the system. These concepts serve as essential building blocks for understanding the complexities of the system and informing decision-making processes aimed at optimizing performance and achieving desired outcomes. The initial pathway analysis reveals a fully connected relationship among “X4,” “X1,” and “X11.” Moreover, it indicates that the identified paths involving these factors exhibit a higher level of connectivity compared to other pathways within the system. Understanding and leveraging these connections are crucial for comprehensively addressing the dynamics and optimizing the performance of the system.

**Table 11:** Paths and the most important concepts concatenation.

Path	Most effective concepts	Indirect effect	concepts concatenation	Target concept
1	X1	0.994	X4, X11	Team cognition (X14)
2	X2	0.996	X4, X11	Team cognition (X14)
3	X6	0.990	X4, X11	Team cognition (X14)
4	X10	0.985	X4, X11	Team cognition (X14)
5	X12	0.993	X7, X11	Team cognition (X14)

## 4 Discussion

The study aimed to identify and explain the factors that influence team cognition within ERTs by constructing a comprehensive model. This model highlights the relative importance, hierarchical structure, and cause-and-effect relationships among these factors. The research also examines the interactions between these factors in emergency contexts. To achieve its objectives, the study utilized a multi-faceted approach that integrates various analytical techniques, including meta-synthesis, the BWM, ISM, and FCM.

The study identified TCSFs affecting ERTs through a thorough literature review across multiple sources. A seven-stage meta-synthesis model was employed to systematically extract and categorize the TCSFs into 13 distinct categories. To ensure reliability, the study used the CASP tool to assess content quality, and the researcher’s evaluations were cross-validated with those of a field expert. The Kappa coefficient was also calculated to measure inter-rater agreement, reinforcing the accuracy of the extracted concepts. With a Kappa value of 0.63, surpassing the acceptable threshold of 0.6, the reliability and validity of the extracted concepts and their categorization were confirmed as acceptable. Team cognition refers to the organized cognitive structures that enable team members to share, store, and retrieve both individual and collective knowledge while interacting with one another (Tasca, 2021). This collective cognitive process is essential for effective collaboration and decision-making, especially in dynamic and complex environments like emergency response situations. Numerous studies have shown a strong correlation between team cognition and team performance, emphasizing the need to understand their relationship (Fernandez et al., 2017; Zhao et al., 2023). Team cognition includes the combined expertise, skills, experiences, and information of team members, while team performance measures how effectively and efficiently teams achieve their objectives (Zhu and Wholey, 2018). Identifying and assessing the factors that influence team cognition is vital for enhancing team performance (Wooldridge et al., 2024). By understanding these key determinants, organizations can implement targeted interventions to improve team dynamics, decision-making, and overall effectiveness. Previous studies by Laurila-Pant et al. (2023) and Meng et al. (2022) have contributed to the understanding of factors influencing shared situational awareness in specific domains, like disaster simulations and flight crew operations, identifying important elements such as communication, standards, decision-making, safety culture, and training. However, these studies may lack the comprehensiveness of the present research. By employing a holistic approach and integrating multiple analytical methods, this study aims to identify, prioritize, and analyze a broader range of factors affecting team cognition in emergency response scenarios. These studies are nonetheless narrow and shallow compared to the comprehensiveness of the present research. The study will adopt a holistic approach, integrating different analytical methods in the identification, prioritization, and analysis of a wider range of factors that influence team cognition during emergency responses. This provides a nuanced understanding of such factors, hence making them applicable in a wide variety of emergency contexts. These findings have important implications for practice, given that they allow the design of specific interventions focused on improving critical success factors of ERT performance. This study fills the gap in the existing literature and offers some practical implications to help improve the current understanding of team cognition in emergency response settings.

The Best-Worst Method (BWM) was used to assign weights to various factors identified in the study, revealing that “Team maturity (The team members harmonization)” and “Inefficient 4Cs (communication, coordination, cooperation, and collaboration)” have the most significant impact on team cognition and overall performance of Emergency Response Teams (ERTs) during emergencies. Team maturity encompasses elements that foster harmony and cohesion among team members, including shared understanding, trust, effective interaction, and aligned priorities. Heldal et al. (2020) emphasized the importance of joint understanding among intercultural team members for enhancing shared cognition. Additionally, McNeese and Reddy (2017) highlighted the dynamic nature of team cognition and the pivotal role of shared understanding in its development. Tasca’s review identified trust among team members as a crucial factor in enhancing team cognition, calling for further research in this area (Tasca, 2021). Various studies consistently demonstrate that teams with high interaction levels among members tend to perform better in cognitive processes and overall effectiveness (Cooke et al., 2007; Cooke, 2015; Cooke et al., 2023). Furthermore, research by Franke et al. (2021)indicates that goal alignment significantly enhances team decision-making and overall performance, while Niler et al. (2021) found that stronger perceptions of cognitive alignment among team members correlate with improved performance. Some other forms of intervention include team building, communication training, and the establishment of the element of trust among teammates that may also allow practical development strategies in regard to this insight into play. Training through scenario-based methodical procedures for emergencies shows results in increasing teams’ coordination levels. For these practical measures target the actual reasons and give impetus to greater teams for awareness as well as results at real incidents of emergencies.

Another significant factor influencing team cognition identified in this study is the concept of “Inefficient 4Cs.” This factor has been emphasized in several studies for its critical role in team dynamics. For instance, Mahmood et al.’s (2022) findings highlight the interconnectedness between shared cognition and intra-team communication and their combined impact on team effectiveness. Kennedy and McComb’s (2010) research elucidated the role of verbal communication and coordination in facilitating the macrocognitive process of mental model convergence. Similarly, Fiore et al.’s (2010) study highlights the pivotal role of understanding collaboration in optimizing cognitive processes within teams. Furthermore, Nonose et al. (2012) demonstrated the significant influence of metacognition on cooperation and its importance for fostering positive teamwork dynamics. Improving team cognition requires addressing 4Cs inefficiencies, as these findings show. These inefficiencies can be directly addressed through practical steps like implementing better communication tools, team coordination training, and collaborative decision-making frameworks. For example, using real-time communication during emergency simulations enhances coordination and teamwork in stressful situations. To enhancing 4Cs efficiency, improving team macrocognition, facilitating organizational goals such as adaptability and faster decision-making in dynamic contexts are useful. Overall, these findings can help us develop effective strategies to improve the team cognitive abilities and performance of ERTs.

The study applied ISM after the BWM analysis to establish a hierarchical structure among identified factors influencing ERTs. “Improper team training programs” was identified as the most critical factor affecting cognitive performance, highlighting the importance of effective training for enhancing team preparedness and decision-making during emergencies. The MICMAC analysis further identified four shaping factors—“Using technology and tools,” “Lack of procedures or incomplete procedures of teamwork,” “Leadership behavior and performance,” and “Monitoring the performance of the emergency response team”—which significantly influence other factors in the system. In a study by Meng et al. (2022) that aimed to identify the hierarchy of factors influencing TSA in flight crews, a similar approach to the present study was utilized, employing the ISM method. Both studies identified factors related to training and policy (or procedure) as fundamental elements positioned at the root layer of the hierarchy. Other research in team cognition has also highlighted training as a crucial factor; for instance, Salas et al. (2007) emphasized the importance of both formal and informal avenues for developing shared mental models within teams. Grand et al. (2016) pointed out the necessity of investing in training and expertise development to promote knowledge sharing. Additionally, using technology and tools can enhance various facets of team cognition, such as communication and coordination during training and emergency responses. Hadjielias et al. (2021) illustrated how distinct interactions among team cognition, teamwork, and task work shape cognitive states in the digital innovation process. Nunavath et al. (2016) noted that Information and Communication Technology facilitates information distribution and task assignment, improving TSA. Alongside these, in high-pressure environments like emergency response, established teamwork procedures offer structured frameworks, outlining roles and communication protocols that help streamline operations and minimize errors. For example, Fernandez et al. (2017) emphasized the importance of access to procedures and action plans for team duties in healthcare teams. Salas et al. (2007) also introduced “Plan execution” as a key marker of team cognition, underlining its significance in evaluating team performance and effectiveness. These findings show effective ERTs prioritize comprehensive training, advanced technology, and collaborative teamwork. For example, integrating simulated training and real-time performance monitoring enhances team readiness and emergency response. To ensure ongoing ERT effectiveness, organizations need to consistently update and review their procedures guidelines.

Another crucial factor affecting team cognition highlighted in this study is leadership behavior and performance. Leadership significantly influences team dynamics, decision-making processes, and overall effectiveness. Effective leadership fosters a supportive environment for collaboration, communication, and innovation, shaping team members’ cognitive processes and collective outcomes. Toader and Martin (2023) concluded that team leaders’ behavioral preferences have a significant impact on team cognition. Murase et al. (2014) emphasized that leadership behavior plays a critical role in shaping team cognition and driving effectiveness, particularly in multi-team contexts. Santos et al. (2016) suggested that shared temporal cognitions can help mitigate temporal conflict within teams, acting as a substitute for temporal leadership. In addition to effective leadership, monitoring the performance of Emergency Response Teams (ERTs) is vital for shaping team cognition. Systematic performance evaluations, feedback, and a culture of continuous improvement can optimize cognitive processes, coordination mechanisms, and overall team effectiveness. This approach enhances ERTs’ ability to mitigate risks and respond effectively during crises. Salas et al. (2007) highlighted mutual performance monitoring’s key role in fostering effective teamwork, promoting a culture of support, accountability, and continuous improvement. Nonose et al. (2012) indicated that mutual performance monitoring includes assessing individual, partner, or team activities and statuses, demonstrating that equipment analysis could lead to role sharing and adjustments in individual behaviors. Emergency response leadership development programs should be a priority for organizations. The core of these initiatives should empower leaders to cultivate shared understanding, trust, and effective communication under pressure. Real-time feedback tools and comprehensive performance monitoring systems will improve team awareness and responsiveness. Briefly, using tools with immediate feedback during simulations and real emergencies exposes weaknesses, encourages best practices, and enhances team performance. For ERTs, a structured system like an incident command system (ICS) is key to better leadership and performance monitoring. For effective team communication and coordination during emergencies, clear roles, responsibilities, and hierarchies are vital in ICS (Farcas et al., 2021). This framework enhances the focus on responsibilities and alignment with organizational goals by promoting unity of command and reducing uncertainty for leaders and team members. Studies consistently show that ICS enhances decision-making and improves team adaptability under pressure. Integrating ICS principles into training and daily workflows improves team cognition and emergency response efficiency within organizations.

In the final stage of the study, the cause-and-effect relationships between the factors were examined using FCM modeling. The analysis revealed that “Team maturity” and “Inefficient 4Cs” had the highest centrality scores, indicating their pivotal roles in influencing overall team cognition within ERTs. Additionally, the pathway linking “X4,” “X1,” and “X11” was identified as the most critical and impactful for team cognition in ERTs. Leadership, training, and performance monitoring are highlighted in this pathway as interconnected and mutually influential elements that synergistically enhance team cognition. Improving this process significantly enhances team performance and effective decision-making, leadership, and operational readiness during emergencies. These findings show organizations should prioritize interconnected strategies: leadership development, comprehensive training, and systematic performance monitoring. For example, combining leadership simulations and feedback-driven training modules can improve teamwork, creating a more unified and effective emergency response team for high-pressure situations.

The subjectivity inherent in expert judgments and pairwise comparisons influences this study’s findings. While the BWM minimizes inconsistencies in expert opinions through optimized comparison ratios, a low inconsistency ratio of 0.067 still suggests minor data inconsistencies. Bias in evaluating team cognition factors could stem from the experts’ professional backgrounds and experience. For instance, whereas scholars focus on theoretical issues, emergency responders emphasis practical operations. Although diversity improves the investigation, it also increases the possibility of erroneous conclusions. According to research, expert assessments are impacted by cognitive biases such anchoring and confirmation bias, which can also affect the prioritization of criteria (Kerr and Tindale, 2004). Our findings were supported by the expert panel, who carefully chose members with both academic and practical experience. We chose experts based on their experience and qualifications in team cognition, emergency response, and workplace safety. Also, using a systematic approach, the BWM method reduced inconsistencies by minimizing paired comparisons. The relatively low inconsistency ratio (0.067) indicates a high degree of reliability in the pairwise comparisons made by the expert panel. Rezaei (2016) shows that inconsistency ratios under 0.1 yield dependable conclusions. It is important to acknowledge that even minimal inconsistencies can affect the results. Despite using methods like BWM and FCM to reduce inconsistencies, some subjectivity from expert input will always remain. To validate and enhance our findings, this limitation shows we must confirm expert opinions with empirical data or different approaches, for example, structured interviews using grounded theory.

The study’s reliance on experts also limits the generalizability of its findings, as the conclusions are context-dependent and reflective of the specific domains and professional backgrounds of the consulted experts. To address this limitation, future research could incorporate broader and more diverse expert panels and employ field studies, such as emergency response drills and simulations. The reliability and applicability of the results are enhanced by these practical exercises, which offer real-world insights through observation and analysis of team cognition. Furthermore, real-world application of these findings can improve team cognition during emergencies. For example, organizations might develop focused training to build shared understanding, enhance communication, and improve teamwork during stress. Teams can utilize customized scenario-based training, VR simulations, and tabletop exercises emphasizing key factors to better predict, adapt to, and react to dynamic emergencies. Furthermore, using feedback loops in drills and post-incident reviews enables ERT members to analyze their cognitive and collaborative work, resulting in enhanced team performance. This research can lead to improved decision support systems and communication protocols by incorporating cognitive and contextual factors, reducing errors, and boosting situational awareness during crises. Future research should investigate individual factors (stress, decision-making, awareness) influencing ERT members’ cognitive performance for a deeper understanding of team dynamics. Through field-based observations and exploration of these dimensions, researchers can develop targeted strategies that improve team cognition and performance in emergencies, resulting in more effective and adaptable ERT operations.

## 5 Conclusion

Identifying and determining factors affecting team cognition is crucial for improving team performance and achieving organizational goals within Emergency Response Teams (ERTs). By investing in the understanding and optimization of team cognition, organizations can unlock their teams’ full potential. This study employs rigorous analysis and modeling to provide insights into the complex dynamics of team cognition in ERTs. Understanding the relative importance of various factors, their hierarchical relationships, and the causal links among them allows stakeholders to make informed decisions and develop targeted interventions to enhance team performance and effectiveness in emergency situations. To improve emergency team cognition, policymakers should prioritize training exercises and scenario-based simulations that translate research findings into practical strategies. In addition, we can develop guidelines for selecting and training team leaders to effectively manage cognitive demands. Public policy can promote the integration of team cognition frameworks into emergency response protocols, encouraging collaboration between industry, academia, and government. This study provides a comprehensive framework for better understanding emergency response and team cognition.

## Data Availability

The original contributions presented in the study are included in the article/Supplementary material, further inquiries can be directed to the corresponding author.
