# Supplementary material for: Unveiling the dynamics of team cognition in emergency response teams
Source: Front Psychol. 2025 Mar 26;16:1534224. doi: 10.3389/fpsyg.2025.1534224 (PMC11979796; doi:10.3389/fpsyg.2025.1534224)
Supplement: Supplementary file 1 [file Table_1.DOCX]

Supplementary Material

# Supplementary Figures and Tables

**Supplementary Table 1.** Team cognition shaping factors based on meta-synthesis analysis

| **New TCSFs** | **Symbol** | **Extracted original sub-factors (first-level codes)** |
| --- | --- | --- |
| Team maturity (The team members harmonization) | X1 | Divergent priorities at the scene interoperability risks (1). team can fail if interactions are lacking or ineffective coordination occurs (2). team cognition aligning with joint action of team members (2). Effective emergency cooperation requires better interaction processes. (3). Team task confidence positively impacts team performance, (4). The cognitive process needs to be shared for a common understanding of the ongoing situation (5). The scenario distinctly deviated from everyday situations (6). Pre-establishing core team members in an Incident Management Team (IMT) enhances initial trust (7). The data suggests an inverse correlation between team interaction levels and team performance scores (8). team processes might mediate the relationship between taskwork and teamwork similarity and team performance (9). Cognition in crisis management teams requires analyzing interactions among teams. (10). To measure team cognition, the focus is on defining interactions both within and across the cognitive systems (11). Better team performance links to member familiarity; time crucial for meta-knowledge retrieval (12). shared beliefs among team members enhance adaptability to the environment (13). Similarity index measures alignment of beliefs; excessive confidence and fixation hinder team understanding (14). Team members benefit from shared understanding of tasks, roles, and expertise (15). High interaction and trust lead to sharing specialist knowledge (16). Teams develop shared goals, norms, and align individual roles with objectives. (17). Evaluating team effectiveness involves assessing shared knowledge and understanding. (18). Shared familiarity with roles and responsibilities promotes team understanding (19). A well-designed heterogeneous team enhances team performance (20). Inter-cluster interactions facilitate quick consensus attainment among large groups (21). Bureau-political interactions often facilitate rapid, fragmented crisis responses (22). Trust and effective communication depend on understanding past relationships (23). Personnel in multi-agency operations interpreted incidents differently (24). Ensuring a shared understanding of the situation, priorities, and actions is crucial (25). Comprehend the interface between teams in a multi-organizational context (26). Team members are striving towards a shared goal (27). Team members intentionally consider their actions' impact on teammates (28). Crisis teams excel when members prioritize teamwork and common goals (29). Common Operating Picture (COP) serves as the foundation for decision-making in IMTs. (30). Resilience is a crucial characteristic of a Joint Cognitive System (JCS) (31). Cross-level interactions are vital for the inter-agency information sharing system to function (32). Teams improve incrementally with more autonomous agents (33). Team members should memorize the responsibilities of each assistance unit (34). COP relies on mutual trust among agencies (35). Liaison officers, may have established trust, enabling effective dialogical communication (36). Group coordination evolves as roles adapt to conflicts (37). Identifying system trust components guides search and rescue (SAR) organizations in development (38). High SA requires role understanding and organizational awareness during disasters (39). Heedful interrelating and sense-making are crucial in disaster decision-making (40). Human-centered technology design prioritizes team interaction (41). Information relevance depends on understanding different roles (42). Team performance relies on diverse expertise, clear roles, and efficient tasks (43). Team effectiveness linked to abandoning entrenched interaction pattern (44). Team interaction complexity influences task complexity (45). Visualizing interaction networks among emergency teams (46). Enhance interaction between executive and first responder teams (47). Individual and team involvement within the network improved (48). Enhance crisis management team interactions (49). Team cognition encompasses intertwined individual and team-level processes (50). |
| Inefficient 4Cs (communication, coordination, cooperation and collaboration) | X2 | Implementation of strategic goals enhances communication among agencies (51). Verbal communication crucial for assessing sensemaking in macro-cognition (52). Emergent MTS structures disrupt coordination (53). Weak user cooperation hampers disaster management (54). Verbal communication greatly impacts team SA (55). Command struggles to communicate with field teams for updates (56). Trust issues hinder communication and collaboration (5). Actors focused on communicating decision-relevant information (6). Time pressure boosts dyadic communication (57). Communication skills enable members in improvisation (58). Accurate mental representations enhance team coordination (13). face-to-face communication (59). Sender's communication affects recipient's attention to information (60). Multiteam communication processes reveal more uncertainty affecting SA (19). Enhanced information sharing observed when emergency responders collaborate (61). Reminder of designated roles and responsibilities in collaborative activities (62). Team communication styles influence diagnosis performance (63). Coordinator-facilitated information sharing enhances SA (64). Short-term coordination faces on-site communication challenges (65). Shared Mental Models (SMM) are affected by communication modes (23). Implicit coordination better than verbal communication for teamwork (66). Verbal updates had the potential to be highly beneficial (25). through communication, team forms a shared team world information (27). Implicit coordination prevailed in communication during the SAR flight (28). Coordination challenges linked to weak shared mental models. (67). Assertive yet cooperative approach best for crisis decision-making (29). Coordinating decision-making within an IMT (30). Improving decision-making through precise, timely communication (68). Team cognition is accelerated by communication (33). Agent connections depict the flow of information within the system (35). Collaborating with emergency center crucial for addressing uncertainties (36). communication, coordination, and control are interdependent processes (69). Developing communication processes can be based on critical information (38). (53) Team SA in disasters relies on effective communications. (39). To get crucial info from teammates, choose the right way to communicate (41). Make information actionable for users in emergency response (ER) coordination (42). coordination focuses on organizing and distributing tasks among team members (70). Coordination among organizations is vital for crisis and emergency management (47). Network connectedness assesses emergency coordination potential (48). Team cooperation crucial for effective crisis management. (49). Teams showed notable communication restructuring (50). |
| Using technology and tools | X3 | Communication technology improve ER and its simulation (71). using the spreadsheet artifact has enhanced decision-making effectiveness (54). To promote a shared mental model in remote communication, utilize both offline and online technologies (56). Maps were employed to enhance SA, communication, and decision-making (6). Effective decision supports for IMTs aid in representing and managing pertinent information (7). Improved displays that spatially engage teams, can enhance team performance (8). CMT members interact dynamically, using tech tools as needed (11). Automating critical coordination communication with meta-knowledge via software agent (12). Technology could also contribute to enhancing resilience (58). Leveraging technology can enhance information-sharing processes (60). Handheld radios facilitate communication across dispersed sites (19). Technology enhances information sharing by virtually co-locating emergency responders (61). The multiple-view, role-based design aids teams in managing their information (62). Deep learning and defined rewards can enhance shared beliefs (20). Mutual awareness tool in interfaces enhances team members' information access (63). Ensure voice communication infrastructure allows easy access to various channels like radio, mobile, and landline (72). Users connect socially around the uTable using verbal and facial expressions (66). Shared displays and information logs can help with information sharing (25). Crisis decision tools aid in information sharing, knowledge retrieval, and negotiation (29). IMTs integrate inputs into the COP using diverse platforms (30). An information management solution, aids in locating and sharing information (73). Information Technology plays a crucial role in building SA (39). Human-centered Technology design (41). Text-based tech vital in emergencies (70). Current collaborative platforms lack support for advanced activity awareness (43). |
| Failure in decision-making | X4 | Barriers hinder decision-making in multiteam systems (51). Disorderly decisions on Multiteam System formation impacted subsequent processes (53). Teams with adequate intellectual resources may compensate for fatigue in performance (74). Operational decisions rely on SA created by response agents (5). ER scenarios have limited time for decision-making (6). Firefighting and incident command are stressful, affecting individual performance (7). Timeliness is crucial for effectiveness in modern organizations (9). Teams with meta-knowledge enhance members' cognitive capacity for tasks (12). In high-stress conditions, participants felt overwhelmed by workload (57). Task complexity influenced shared beliefs in certain teams (13). Teams handle uncertainty and anxiety from nonroutine events (15). Spatial information played a crucial role during critical decision points (75). Network teams excel in making correct decisions in complex environments (16). Shifting focus to interpretation of information can overcome stagnation in decision-making (76). High-performing teams share structured information for faster decisions (77). Dynamic teams handle time pressure, complexity, and task risks (18). Time pressure in extreme environments requires careful decision management (60). Fire and ambulance teams faced time pressure to assist train casualties (19). Team risk tolerance boosts success in uncertain environments (20). More widely accepted decision agents hinder preference convergence (21). On-site coordinators made crucial decisions with limited communication and information (65). The complexity itself poses a potential challenge to SMM (23). SAR operations allocated limited resources dynamically for critical cases (30). Decision-making evolved in meetings, reflecting major incident stages (78). Actor's skill in addressing dynamic threats is crucial in disaster response (40). Well-informed decisions prevent later problems and save time in response operations (42). Decision-making wheel model from team cognitive work analysis (45). Increased transitions may indicate critical decision points (79). stress and fatigue effects on decision-making in abnormal situations (80). The team faced challenges in deciding on the right course of action (50). |
| Improper team training programs | X5 | Clear goals in ER stress the need for thorough training (51). Goal-oriented training enhances explicit planning, SA, and decision-making (81). Navigating challenges involves organizing drills effectively (71). Exercise scenarios prioritize assessing data acquisition fluency (5). Training can expedite skill acquisition through experience (7). IMT training should integrate self-organizing interaction patterns and adaptive roles (10). Teams acquiring meta-knowledge develop an effective schema (12). Bystanders without emergency room training hindered operations at the crash site (82). Training enables a crucial distinction in same-role improvisation (58). Training helps team members mitigate information complexity effects (13). Similarity index differences inform training and structural changes in teams (14). Interventions might focus on overlearning specific routines for effective adaptation (15). Training team members on responsibilities fosters shared situational understanding (18). Implementing communication-focused training programs is beneficial (60). Emergency responders, trained in managing smaller-scale incidents (19). SMM should be applied throughout training and exercises (23). Operational limitations are linked to the practical challenges of cross-training (28). Regular training enhances team decision-making during crises (29). Role-switching in exercises gives team members diverse experience (34). training should focus on swiftly identifying volunteers' willingness, tasks, and needed support in emergent groups (37). Improved sense-making training enables a more open decision-making process (40). Cross-training prepares players for diverse roles and builds SMMs (41). Reflexive training requires rethinking operational education (42). SMM researchers analyzed and criticized training protocols (43). Findings have big implications for crisis training (44). Simulation informs training for pressured decision-making groups (79). Emergency training exercises help participants focus on various concerns (49). Develop tailored training programs for teams (80). Team members underwent simulation training to identify reorganization mechanisms (50). |
| The quality of information and information sharing process | X6 | In unclear, non-urgent decisions, inter-agency communication decreased, focusing on intra-agency information sharing (51). Close connection of information processing with Leader airtime management in the team (55). Field teams should avoid irrelevant information to prevent distraction or mental overload (56). Information systems generate COP, offering real-time or spatio-temporal information (5). Actors need to grasp relevant information and its relation to goals (6). key IMT members must acquire, represent, and distribute information effectively (7). Diagnosing in crisis management involves assessing vulnerability with incoming information (11). The quality of information is vital for supporting SA (82). The more information requests, the lower the task score (57). More shared information means better SMM and SA (14). Concept mapping for teams to share and integrate vital information (15). Spatial information's impact on team cognition varies with team composition (75). Lack of a common ICT infrastructure and physical proximity hindered information sharing (83). Network structures enable rapid information exchange within teams (16). The information-sharing infrastructure is sufficient for sharing factual information (76). Teams consistently share information about each member's planned actions (18). Both sender and recipient can adopt behaviors to improve access to relevant information (60). Standard data frameworks show when decentralized structures improve information sharing (19). Experts stressed physical co-location as crucial for improving information exchange (61). Timely access to relevant information is vital for understanding dynamic situations (84). Enriched information provision is a way to enhance situation awareness (64). Extreme operating conditions result in information limitations (65). Sensemaking theory examined the impact of unclear early information on actions and understanding (72). A large-scale display offers increased information (66). The information flow's capacity and speed are improved (66). Overloaded telephone lines hindered information confirmation (25). Information is spread among team members (27). Messages relayed through extensive disaster management networks (67). Information sharing processes need better mechanisms to avoid filtering out relevant information (32). Decision makers share SA, creating a final Shared SA for all (85). Team members must recognize all available relevant information (34). Increasing need to gather information about immediate consequences (78). COPs concept provides agents with necessary information, avoiding overload (35). The critical information needs can identify the information requirements (38). Information quality aspects ought to be designed to support a variety of information (73). Key decisions in the game involve choosing and sharing information (41). Interpretation of information is crucial for coordination (42). The information provided encompasses situational details, procedures, and feedback (70). Inconsistent information between control center and failure district stations (45). Repeated requests for information could be viewed as decision inertia or exploration (79). ER agencies need this information for critical response tasks (49). |
| Team members incorrect sensemaking of the crisis | X7 | Sensemaking involves grasping crises, their consequences, and required actions (52). Disaster sensemaking is Interpreting information for understanding. (5). Diverse perspectives crucial for collective sensemaking (11). Dyad demonstrated improved sense-making, implying difficulty in handling multiple events (57). High-performing teams actively participated in extensive collective sensemaking (77). Teams may face challenges due to inadequate sensemaking (86). Sense-making involves making assumptions and attributing significance (32). Neglecting shared sensemaking leads to issues later in the response operation (42). Sensemaking and sensegiving heightened with increased ambiguity (87). |
| Lack of procedures or incomplete procedures of teamwork | X8 | Lack of clear information led teams to rely on outdated routines or improvise (53). Teams should incorporate a brief "how are we doing as a team?" assessment into IMT briefings. (7). CMTs are expected to fulfill their roles outlined in guidelines (10). Untrained bystanders at the crash site lacked knowledge of standard procedures (82). Limited planning for emergency procedures (84). Providing individual and team maps to aid information sharing (62). Rules and procedures serve as vital information for a shared understanding (28). IMTs innovate when plans fail under overwhelming demands (30). A checklist aids in sharing task division knowledge among team members (34). Standard procedures focus information for specialized response teams (35). Overemphasizing standardized procedures can hinder less effective crews (44). Simulation findings guide future policies for pressured decision-making teams (79). Precise rules and regulations are crucial for enhancing performance (47). Clear plans and instructions are vital for ER coordination. (48). Clear rules minimize stress and fatigue (80). |
| Team cultural, contextual, organizational and social conditions | X9 | Variability in team cognitive activity cannot be separated from its context (2). Organizational culture differences impact the sense-making process (3). Cultural factors influencing people's adherence to rules and instructions (71). User context awareness crucial for effective disaster management (54). Field teams need adaptable information systems for context-based data adjustments (56). Socio-cultural factors influence ERT performance (5). Social contexts influenced participants' roles and interactions in the SAR exercise (6). Highly skilled IMT members are crucial for effective operations (7). Diverse CMTs leverage varied backgrounds for comprehensive perspectives (11). Context shapes Situational Awareness. (82). Positive team mood boosts anticipation and awareness (57). Network teams respond faster than hierarchical teams (16). Assessing behaviors in extreme contexts (19). Decision-making groups in dynamic environments must recognize high-risk factors (21). The experimenter noted communication style differences among teams (63). Organizational interests align in collaborative decision-making (22). Effect of operational context on managerial performance (65). Responding organizations had different priorities and decision cues (24). Frameworks analyze system impacts on teamwork in specific contexts (26). Crisis decision-making is akin to bureaucratic negotiation context (29). Sensemaking revolves around contextual rationality (86). Groups showed consistent distribution but significant activity transition differences (78). Studying communication types and social contexts proved useful (38). Social capital relates to the collective impact of member actions (43). Composition of multi-agency groups responding to major incidents in the UK may vary (79). Emergency Operations Center discussions involve sharing team members' perspectives and concerns (49). Experienced teams executed timely and effective reorganizations (50). |
| Actual situation knowledge (or awareness) and perceived situation knowledge (or awareness) | X10 | Positive effect of Knowledge and situation awareness on performance (4). Awareness of data and its sources affects fluency and challenges (5). Team knowledge and interaction negatively influenced team situational awareness (8). Diagnosing involves collaboratively characterizing perceived knowledge (11). Teams with meta-knowledge outperform those without it (12). Local knowledge of team members could aid in locating the crash site (82). Domain knowledge is effective in team members' improvisation (58). Interventions focus on quick and practical knowledge and skills without elaborate details (15). Spatial awareness boosts human-agent team cognition in human-agent teams (75). In network conditions, specialists transferred more Al Qaeda knowledge to non-specialists (16). Early team transitions promote knowledge sharing and improve task execution (17). Multidisciplinary teams improve by working together to build knowledge (18). Role knowledge crucial for deciding what to share (61). Mutual awareness enhances team diagnosis (63). Rescue operation faced coordination challenges due to limited situational awareness (65). Improve sensemaking by addressing "knowing where" and "knowing what" concerns (72). Past experiences shape present actions (27). Not everyone needs to know everything in diverse teams. (28). Prioritize similar situation models early in cooperation (34). Cooperation roles awareness boosts communication (38). Local experts should leverage their shared environmental knowledge (40). Situation awareness supports effective crisis response teams (41). Officers acknowledge each other's expertise and roles differently. (42). Examination of potential activity awareness issues and proposed solutions (43). |
| Leadership behavior and performance | X11 | Less effective team leaders used more opening statements during initial decision-making (55). Leaders using Recognition-Primed Decision (RPD) rely on experience for decision-making (5). Leadership impacts team members' mental models (13). Over time, team members' beliefs converge with the chief of staff's beliefs (14). Hierarchical teams spend more time due to the leader's decision-making process (16). Initial info-sharing issues across command levels impacted Tactical, delaying vital info identification (60). The fire tactical commander developed a plan for evacuating casualties (19). Strong leadership is vital for agenda focus (84). balancing competing values by operational leaders (22). Managerial roles may need restructuring to maintain coordination (65). The final characteristic is shared leadership, exemplified in the SAR team (28). A team leader can assist team members (34). Leader's ability to provide clear direction and lead the team (70). Crisis management leadership with multiple lead organizations faces significant challenges (47). Crew leaders frequently guided sensemaking and sense giving efforts (87). |
| prior knowledge, mental models and transactive memory system | X12 | ER is marked by ambiguity due to lack of prior knowledge and mental models (5). Temporal Team Mental Models were assessed using two distinct methods, capturing shared knowledge over time (9). Findings have implications for improving team memory and knowledge accuracy (12). Team membership is a predictor of more accurate mental representations (13). Team members learn together, improving shared task understanding and transactive memory system (15). Spatial information increased mental model similarity in teams (75). Teams with similar mental models develop clearer action patterns over time. (17). Face-to-face interactions and learning in diverse teams build shared understanding and expertise exchange (59). Shared mental models are crucial components of emergency management (23). Emergency responders need clear, shared mental models for effective emergency management (26). Team decision-making in crises benefits from an integrative mental model (29). Team members exhibited shared mental models by grasping each other's perspectives. (41). In transactive memory, shared mental models are crucial for comprehensive understanding (43). |
| Monitoring the performance of the emergency response team | X13 | The monitoring protocol helps improve disaster responders' performance. (5). Monitoring IMT processes crucial for identifying and fixing issues (7). Real-time monitoring triggers help for distressed team members (57). Network structures aid teams in monitoring each other's performance (16). Monitoring team member communications and explicitly providing crucial information(84). Monitoring decision-making speed versus careful consideration is useful. (22). Teams monitoring complex systems mainly perform routine tasks (44). Continuous monitoring reduces stress and fatigue (80). |

References:

1. Cattermole-Terzic V, Horberry T. Improving traffic incident management using team cognitive work analysis. Journal of Cognitive Engineering and Decision Making. 2020;14(2):152-73.

2. Cooke NJ, Gorman JC, Myers CW, Duran JL. Interactive team cognition. Cognitive science. 2013;37(2):255-85.

3. Giordano R, Pagano A, Pluchinotta I, del Amo RO, Hernandez SM, Lafuente ES. Modelling the complexity of the network of interactions in flood emergency management: The Lorca flash flood case. Environmental Modelling & Software. 2017;95:180-95.

4. Hamilton K, Mancuso V, Mohammed S, Tesler R, McNeese M. Skilled and unaware: The interactive effects of team cognition, team metacognition, and task confidence on team performance. Journal of Cognitive Engineering and Decision Making. 2017;11(4):382-95.

5. Laurila-Pant M, Pihlajamäki M, Lanki A, Lehikoinen A. A protocol for analysing the role of shared situational awareness and decision-making in cooperative disaster simulations. International journal of disaster risk reduction. 2023;86:103544.

6. Luokkala P, Virrantaus K. Developing information systems to support situational awareness and interaction in time-pressuring crisis situations. Safety science. 2014;63:191-203.

7. McLennan J, Holgate AM, Omodei MM, Wearing AJ. Decision making effectiveness in wildfire incident management teams. Journal of Contingencies and Crisis Management. 2006;14(1):27-37.

8. McNeese NJ, Schelble BG, Canonico LB, Demir M. Who/what is my teammate? Team composition considerations in human–ai teaming. IEEE Transactions on Human-Machine Systems. 2021;51(4):288-99.

9. Mohammed S, Hamilton K, Tesler R, Mancuso V, McNeese M. Time for temporal team mental models: Expanding beyond “what” and “how” to incorporate “when”. European Journal of Work and Organizational Psychology. 2015;24(5):693-709.

10. Moon J, Sasangohar F, Peres SC, Son C. Naturalistic observations of multiteam interaction networks: Implications for cognition in crisis management teams. Ergonomics. 2023(just-accepted):1-27.

11. Moon J, Sasangohar F, Son C, Peres SC. Cognition in crisis management teams: An integrative analysis of definitions. Ergonomics. 2020;63(10):1240-56.

12. Müller R, Graf B, Ellwart T, Antoni CH. How Software Agents Can Help to Coordinate Emergency Response Teams: Adaptive Team Performance Comparing Manual and Automated Team Communication. Journal of Business and Psychology. 2023;38(5):1121-37.

13. Sætrevik B. Psychophysiology, task complexity, and team factors determine emergency response teams’ shared beliefs. Safety science. 2015;78:117-23.

14. Sætrevik B, Eid J. The “similarity index” as an indicator of shared mental models and situation awareness in field studies. Journal of cognitive engineering and decision making. 2014;8(2):119-36.

15. Santos CM, Uitdewilligen S, Passos AM, Marques-Quinteiro P, Maynard MT. The effect of a concept mapping intervention on shared cognition and adaptive team performance over time. Group & Organization Management. 2021;46(6):984-1026.

16. Schraagen JM, Veld MHit, De Koning L. Information sharing during crisis management in hierarchical vs. network teams. Journal of contingencies and crisis management. 2010;18(2):117-27.

17. Uitdewilligen S, Rico R, Waller MJ. Fluid and stable: Dynamics of team action patterns and adaptive outcomes. Journal of Organizational Behavior. 2018;39(9):1113-28.

18. van der Haar S, Segers M, Jehn K, Van den Bossche P. Investigating the relation between team learning and the team situation model. Small Group Research. 2015;46(1):50-82.

19. Waring S, Alison L, Shortland N, Humann M. The role of information sharing on decision delay during multiteam disaster response. Cognition, Technology & Work. 2020;22:263-79.

20. Wu H, Ghadami A, Bayrak AE, Smereka JM, Epureanu BI. Impact of heterogeneity and risk aversion on task allocation in multi-agent teams. IEEE Robotics and Automation Letters. 2021;6(4):7065-72.

21. Yin X, Xu X, Chen X. Risk mechanisms of large group emergency decision-making based on multi-agent simulation. Natural Hazards. 2020;103:1009-34.

22. Kalkman JP, Kerstholt JH, Roelofs M. Crisis response team decision‐making as a bureau‐political process. Journal of Contingencies and Crisis Management. 2018;26(4):480-90.

23. Carter E, French S. Are current processes for nuclear emergency management in Europe adequate? Journal of radiological protection. 2006;26(4):405.

24. McMaster R, Baber C. Multi-agency operations: cooperation during flooding. Applied ergonomics. 2012;43(1):38-47.

25. Militello LG, Patterson ES, Bowman L, Wears R. Information flow during crisis management: challenges to coordination in the emergency operations center. Cognition, Technology & Work. 2007;9:25-31.

26. Owen C, Bearman C, Brooks B, Chapman J, Paton D, Hossain L. Developing a research framework for complex multi–team coordination in emergency management. International Journal of Emergency Management. 2013;9(1):1-17.

27. Plant KL, Stanton NA. All for one and one for all: Representing teams as a collection of individuals and an individual collective using a network perceptual cycle approach. International Journal of Industrial Ergonomics. 2014;44(5):777-92.

28. Plant KL, Stanton NA. Distributed cognition in Search and Rescue: loosely coupled tasks and tightly coupled roles. Ergonomics. 2016;59(10):1353-76.

29. Van Santen W, Jonker C, Wijngaards N. Crisis decision making through a shared integrative negotiation mental model. International Journal of Emergency Management. 2009;6(3-4):342-55.

30. Son C, Sasangohar F, Peres SC, Moon J. Muddling through troubled water: resilient performance of incident management teams during Hurricane Harvey. Ergonomics. 2020;63(6):643-59.

31. Son C, Sasangohar F, Peres SC, Neville TJ, Moon J, Mannan MS. Modeling an incident management team as a joint cognitive system. Journal of Loss Prevention in the Process Industries. 2018;56:231-41.

32. Bharosa N, Lee J, Janssen M. Challenges and obstacles in sharing and coordinating information during multi-agency disaster response: Propositions from field exercises. Information systems frontiers. 2010;12:49-65.

33. Schelble BG, Flathmann C, McNeese NJ, Freeman G, Mallick R. Let's think together! Assessing shared mental models, performance, and trust in human-agent teams. Proceedings of the ACM on Human-Computer Interaction. 2022;6(GROUP):1-29.

34. van der Haar S, Li J, Segers M, Jehn KA, Van den Bossche P. Evolving team cognition: The impact of team situation models on team effectiveness. European Journal of Work and Organizational Psychology. 2015;24(4):596-610.

35. Baber C, Stanton NA, Atkinson J, McMaster R, Houghton RJ. Using social network analysis and agent-based modelling to explore information flow using common operational pictures for maritime search and rescue operations. Ergonomics. 2013;56(6):889-905.

36. Curnin S, Owen C, Paton D, Brooks B. A theoretical framework for negotiating the path of emergency management multi-agency coordination. Applied ergonomics. 2015;47:300-7.

37. Majchrzak A, Jarvenpaa SL, Hollingshead AB. Coordinating expertise among emergent groups responding to disasters. Organization science. 2007;18(1):147-61.

38. Seppänen H, Mäkelä J, Luokkala P, Virrantaus K. Developing shared situational awareness for emergency management. Safety science. 2013;55:1-9.

39. Son J, Aziz Z, Peña‐Mora F. Supporting disaster response and recovery through improved situation awareness. Structural survey. 2008;26(5):411-25.

40. Takeda M, Jones R, Helms MM. Promoting sense-making in volatile environments: Developing resilience in disaster management. Journal of Human Behavior in the Social Environment. 2017;27(8):791-805.

41. Toups Dugas PO, Kerne A, Hamilton WA. The Team Coordination Game: Zero-fidelity simulation abstracted from fire emergency response practice. ACM Transactions on Computer-Human Interaction (ToCHI). 2011;18(4):1-37.

42. Wolbers J, Boersma K. The common operational picture as collective sensemaking. Journal of Contingencies and Crisis management. 2013;21(4):186-99.

43. Carroll JM, Rosson MB, Convertino G, Ganoe CH. Awareness and teamwork in computer-supported collaborations. Interacting with computers. 2006;18(1):21-46.

44. Stachowski AA, Kaplan SA, Waller MJ. The benefits of flexible team interaction during crises. Journal of Applied Psychology. 2009;94(6):1536.

45. Niu K, Liu W, Zhang J, Liang M, Li H, Zhang Y, et al. A Task Complexity Analysis Method to Study the Emergency Situation under Automated Metro System. International Journal of Environmental Research and Public Health. 2023;20(3):2314.

46. Mohammadfam I, Bastani S, Es-haghi M, Golmohamadi R, Saei A. Assessment of working interactions of emergency team members using social network analysis. Journal of Health and Safety at Work. 2014;4(3):37-48.

47. Abbassinia M, Kalatpour O, Motamedzade M, Soltanian A, Mohammadfam I. Application of social network analysis to major petrochemical accident: interorganizational collaboration perspective. Disaster medicine and public health preparedness. 2021;15(5):631-8.

48. Abbassinia M, Kalatpour O, Motamedzade M, Soltanian A, Mohammadfam I, Ganjipour M, et al. Social network analysis approach to analysis of emergency management team performance. Iran Occupational Health. 2021;18(1):89-101.

49. Bergeron CD, Cooren F. The collective framing of crisis management: A ventriloqual analysis of emergency operations centres. Journal of Contingencies and Crisis Management. 2012;20(3):120-37.

50. Gorman JC, Grimm DA, Stevens RH, Galloway T, Willemsen-Dunlap AM, Halpin DJ. Measuring real-time team cognition during team training. Human factors. 2020;62(5):825-60.

51. Alison L, Power N, van den Heuvel C, Humann M, Palasinksi M, Crego J. Decision inertia: Deciding between least worst outcomes in emergency responses to disasters. Journal of Occupational and Organizational Psychology. 2015;88(2):295-321.

52. Baroutsi N. To observe and evaluate sensemaking: the development of the sensemaking interaction analysis. International Journal of Emergency Management. 2023;18(2):172-201.

53. Fleştea AM, Fodor OC, Curşeu PL, Miclea M. ‘We didn’t know anything, it was a mess!’Emergent structures and the effectiveness of a rescue operation multi-team system. Ergonomics. 2017;60(1):44-58.

54. Ginige A, Paolino L, Romano M, Sebillo M, Tortora G, Vitiello G. Information sharing among disaster responders-an interactive spreadsheet-based collaboration approach. Computer Supported Cooperative Work (CSCW). 2014;23:547-83.

55. Hoven M, Segers M, Gevers J, Van den Bossche P. Leader airtime management and team effectiveness in emergency management command and control (EMCC) teams. Ergonomics. 2023;66(10):1565-81.

56. Jones B, Tang A, Neustaedter C. Remote communication in wilderness search and rescue: implications for the design of emergency distributed-collaboration tools for network-sparse environments. Proceedings of the ACM on human-computer interaction. 2020;4(GROUP):1-26.

57. Pfaff MS. Negative affect reduces team awareness: The effects of mood and stress on computer-mediated team communication. Human Factors. 2012;54(4):560-71.

58. Rankin A, Dahlbäck N, Lundberg J. A case study of factor influencing role improvisation in crisis response teams. Cognition, technology & work. 2013;15:79-93.

59. Van Der Haar S, Jehn KA, Segers M. Towards a model for team learning in multidisciplinary crisis management teams. International Journal of Emergency Management. 2008;5(3-4):195-208.

60. Waring S, Alison L, Carter G, Barrett‐Pink C, Humann M, Swan L, et al. Information sharing in interteam responses to disaster. Journal of occupational and organizational psychology. 2018;91(3):591-619.

61. Waring S, Humann M, Dawson N. Facilitators and barriers to effective information sharing during international disaster response. Journal of emergency management (Weston, Mass). 2019;17(6):469-86.

62. Wu A, Convertino G, Ganoe C, Carroll JM, Zhang XL. Supporting collaborative sense-making in emergency management through geo-visualization. International Journal of Human-Computer Studies. 2013;71(1):4-23.

63. Yuan X, She M, Li Z, Zhang Y, Wu X. Mutual awareness: Enhanced by interface design and improving team performance in incident diagnosis under computerized working environment. International Journal of Industrial Ergonomics. 2016;54:65-72.

64. Van de Walle B, Brugghemans B, Comes T. Improving situation awareness in crisis response teams: An experimental analysis of enriched information and centralized coordination. International Journal of Human-Computer Studies. 2016;95:66-79.

65. Andreassen N, Borch OJ, Sydnes AK. Information sharing and emergency response coordination. Safety Science. 2020;130:104895.

66. Liu J, Qin Y, Yang Q, Yu C, Shi Y. A tabletop-centric smart space for emergency response. IEEE Pervasive Computing. 2015;14(2):32-40.

67. Smith W, Dowell J. A case study of co-ordinative decision-making in disaster management. Ergonomics. 2000;43(8):1153-66.

68. Bearman C, Grunwald JA, Brooks BP, Owen C. Breakdowns in coordinated decision making at and above the incident management team level: An analysis of three large scale Australian wildfires. Applied ergonomics. 2015;47:16-25.

69. Comfort LK. Crisis management in hindsight: Cognition, communication, coordination, and control. Public administration review. 2007;67:189-97.

70. Weger K, Menon V, Hollingsworth A, Schaub H. Information sharing behaviors in text-based communication during emergency response. Journal of Emergency Management (Weston, Mass). 2022;20(6):499-516.

71. Gomes JO, Borges MR, Huber GJ, Carvalho PVR. Analysis of the resilience of team performance during a nuclear emergency response exercise. Applied ergonomics. 2014;45(3):780-8.

72. Landgren J. Supporting fire crew sensemaking enroute to incidents. International Journal of Emergency Management. 2005;2(3):176-88.

73. Seppänen H, Virrantaus K. Shared situational awareness and information quality in disaster management. Safety Science. 2015;77:112-22.

74. Guastello SJ, Correro II AN, Marra DE. Cusp catastrophe models for cognitive workload and fatigue in teams. Applied Ergonomics. 2019;79:152-68.

75. Schelble BG, Flathmann C, Musick G, McNeese NJ, Freeman G. I see you: Examining the role of spatial information in human-agent teams. Proceedings of the ACM on Human-Computer Interaction. 2022;6(CSCW2):1-27.

76. Treurniet W, Wolbers J. Codifying a crisis: Progressing from information sharing to distributed decision‐making. Journal of Contingencies and Crisis Management. 2021;29(1):23-35.

77. Uitdewilligen S, Waller MJ. Information sharing and decision‐making in multidisciplinary crisis management teams. Journal of Organizational Behavior. 2018;39(6):731-48.

78. Wilkinson B, Cohen‐Hatton SR, Honey RC. Decision‐making in multi‐agency groups at simulated major incident emergencies: In situ analysis of adherence to UK doctrine. Journal of Contingencies and Crisis Management. 2019;27(4):306-16.

79. Wilkinson B, Cohen‐Hatton SR, Honey RC. Variation in exploration and exploitation in group decision‐making: Evidence from immersive simulations of major incident emergencies. Journal of Contingencies and Crisis Management. 2022;30(1):82-91.

80. Ávila Filho S, Carvalho A, Portela G, Costa C. Learning environment to take operational decision in emergency situation. Procedia Manufacturing. 2015;3:1780-7.

81. Cohen-Hatton SR, Honey R. Goal-oriented training affects decision-making processes in virtual and simulated fire and rescue environments. Journal of Experimental Psychology: Applied. 2015;21(4):395.

82. O'Brien A, Read GJ, Salmon PM. Situation Awareness in multi-agency emergency response: Models, methods and applications. International Journal of Disaster Risk Reduction. 2020;48:101634.

83. Schraagen JM, van de Ven J. Human factors aspects of ICT for crisis management. Cognition, Technology & Work. 2011;13:175-87.

84. Waring S, Moran JL, Page R. Decision‐making in multiagency multiteam systems operating in extreme environments. Journal of Occupational and Organizational Psychology. 2020;93(3):629-53.

85. Parvar H, Fesharaki MN, Moshiri B. Shared Situation Awareness Architecture (S2A2) for Network Centric Disaster Management (NCDM). International Journal of Computer Science Issues (IJCSI). 2012;9(4):503.

86. Weick KE. The collapse of sensemaking in organizations: The Mann Gulch disaster. Administrative Science Quarterly. 1993;38:628-52.

87. Dwyer G. Enacting safety: Firefighter sensemaking of entrapment in an Australian bushfire context. International Journal of Disaster Risk Reduction. 2022;68:102697.
